# Intramolecular Diels–Alder Reaction of a Biphenyl Group in a Strained *meta*-Quaterphenylene Acetylene

**DOI:** 10.1021/acs.joc.2c02280

**Published:** 2023-01-26

**Authors:** Komal Mittal, Ashley V. Pham, Amanda G. Davis, Abigail D. Richardson, Clement De Hoe, Ryan T. Dean, Vi Baird, Ashley Ringer McDonald, Derik K. Frantz

**Affiliations:** Department of Chemistry and Biochemistry, California Polytechnic State University, 1 Grand Avenue, San Luis Obispo, California 93407, United States

## Abstract

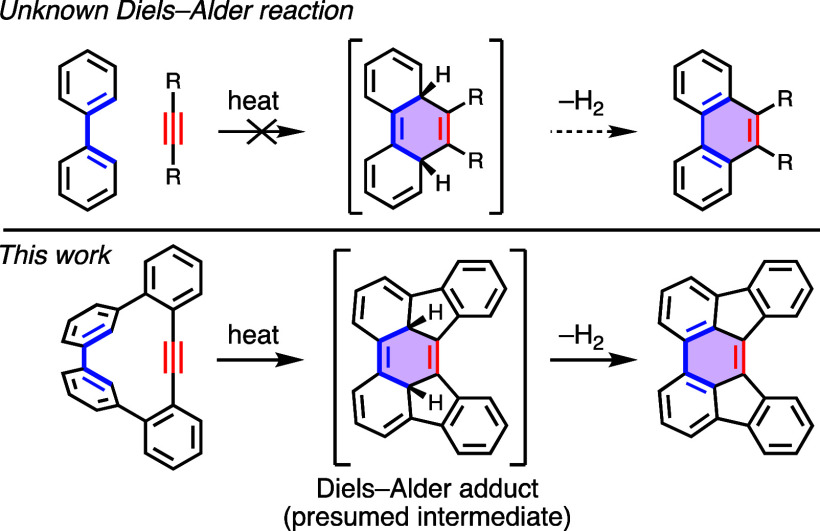

At elevated temperatures, a strained, cyclic *meta*-quaterphenylene acetylene undergoes an intramolecular cyclization reaction to form benz[*e*]indeno[1,2,3-*hi*]acephenanthrylene. This reaction represents an example of a Diels–Alder reaction at the 2-, 1-, 1′-, and 2′-positions of a biphenyl derivative, a region analogous to the bay regions of perylene and other periacenes. The reaction proceeds cleanly with high conversion. Kinetics studies of a methylated derivative reveal that the Δ*G*^‡^ for the reaction is ∼40–41 kcal/mol, and computational models predict a similar value of *G*_rel_ for the transition state of a concerted [4 + 2]-cycloaddition.

The bay regions of perylene and larger periacenes participate in Diels–Alder reactions with dienophiles at elevated temperatures to generate dearomatized adducts, which oxidize to form π-extended structures (Scheme 1).^1^ In 1957, Clar and Zander presented the first report of this type of process—a reaction of perylene ([2,3]-periacene) in molten maleic anhydride with chloranil as oxidant.^2^ Over 50 years later, pioneering work by Scott and co-workers reinvigorated interest in this reaction by demonstrating its utility for extending the π-systems of polycyclic aromatic hydrocarbons (PAHs).^3,4^ Controlled extension of PAHs via Diels–Alder reactions has developed into a synthetically useful technique as a class of the so-called APEX (annulative π-extension) reactions, which have received significant attention over the past decade.^5^

**Scheme 1:**
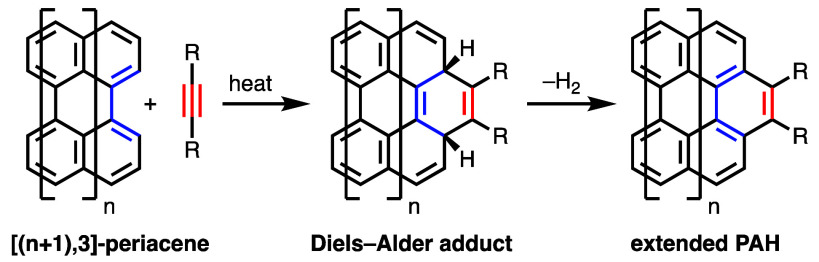
Diels–Alder Reactions of Periacenes with Dienophiles (Alkyne Shown) Generate Extended PAHs Use of brackets to indicate variable sizes of periacenes is inspired by a figure in ref (5).

In 2009, Scott and co-workers reported that perylene (**1**) reacts with excess acetylenedicarboxylate ester **2** over 3 days at 150 °C to form compound **3** (Scheme 2).^3^ The reaction proceeded with under 50% conversion. A larger structure, bisanthene ([3,3]-periacene) derivative **4**, fully reacts with **2** at lower temperature (120 °C) and in less time (1 day) to form compound **5a** and the product of a second addition on the opposite side of the molecule. In a subsequent report, the Scott group showed the remarkable result that compound **4** reacts with unfunctionalized acetylene to form structure **5b**, although with only 21% conversion at 140 °C and 1.8 atm.^4^ Compound **4** also undergoes Diels–Alder reactions to give π-extended products with nitroethylene,^6^ vinyl phenyl sulfoxide (to a lesser extent),^4^ and arynes.^7^ Expanding on the Scott group’s work with perylene, Krompiec and co-workers recently demonstrated that compound **1** reacts with alkyne **2** at 185 °C in *p*-cymene to give structure **3** in 95% yield and that, at 285 °C over 72 h in a vacuum, **1** reacts with diphenylacetylene.^8^ Ajayakumar, Feng, and co-workers recently reported that a derivative of tetrabenzocoronene ([4,3]-periacene) undergoes 2-fold Diels–Alder addition of **2** at 105 °C, in the presence of chloranil, to give the π-extended product in 32% isolated yield.^9^

**Scheme 2:**
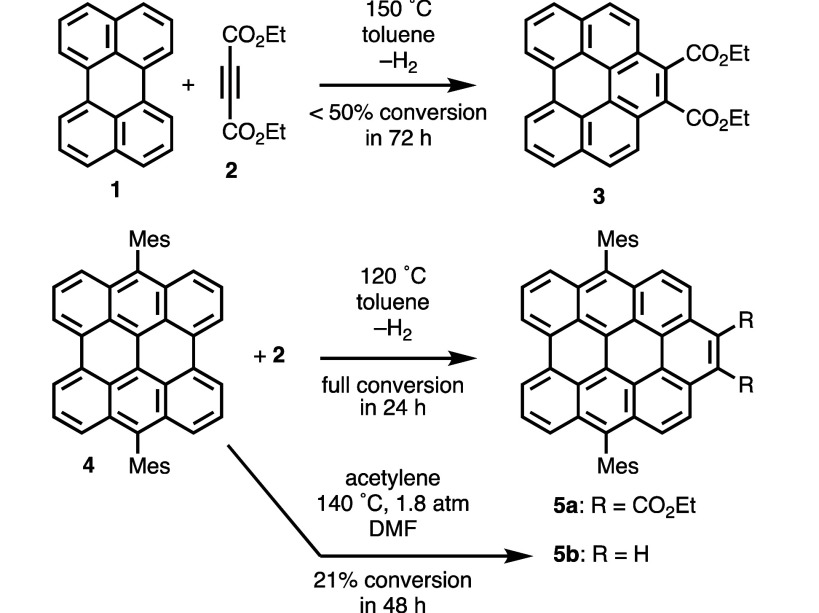
Diels–Alder Reactions of Perylene (**1**) and Dimesitylbisanthene (**4**) Reported by Scott and Co-workers^3^

The experimental observation that reactivity toward Diels–Alder reactions at the bay regions of periacenes increases with extension of the periacene system is unsurprising, because larger systems possess a lower proportion of dearomatized rings in their Diels–Alder adducts, and is supported by computational studies (Figure 1). DFT calculations (B3LYP/6-31G(d)) performed by Scott^3^ indicate that the energetic barriers for Diels–Alder reactions between members of the periacene class with acetylene decrease as the size of the periacene increases (20.9 kcal/mol for tetrabenzocoronene, 24.2 kcal/mol for bisanthene, 30.0 kcal/mol for perylene, and 43.9 kcal/mol for phenanthrene). Using the same method and basis set, Mebel and co-workers calculated that the Diels–Alder reaction of biphenyl with acetylene requires an even higher activation energy (45.2 kcal/mol) than that of phenanthrene.^10^ Fernández and co-workers recently reported a similar trend in their study of the reactivities of periacenes, phenanthrene, and biphenyl toward Diels–Alder reactions with maleic anhydride using sophisticated computational methods (BP86-D3/def2-TZVPP//RI-BP86-D3/def2-SVP).^11^ Not only do phenanthrene and biphenyl require the highest activation energies in their theoretical reactions with maleic anhydride, they are also the only two members of the class whose reactions to the π-extended products, after loss of H_2_, are endothermic.

**Figure 1:**
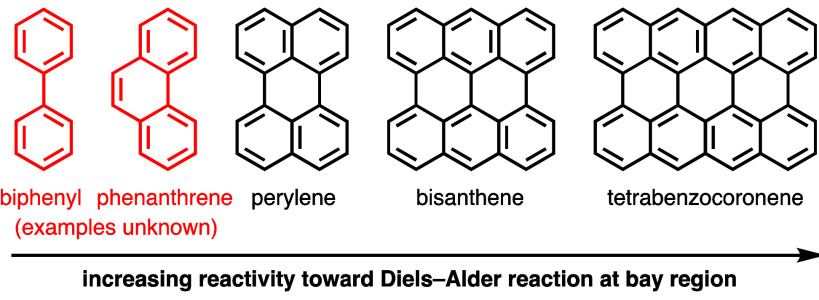
Extension of periacenes increases reactivity toward Diels–Alder reactions at their bay regions. Examples of this reaction are unknown for biphenyl and phenanthrene.

Diels–Alder reactions of the bay region of phenanthrene and the equivalent region of biphenyl (comprising the 2-, 1-, 1′-, and 2′-positions) remain unreported. Carboryne, a powerful dienophile that undergoes [4 + 2]-cycloaddition reactions with toluene and other aromatic compounds, reacts at the 1- and 4-positions of phenanthrene rather than at the bay region.^12^ The strained biphenyl units in cycloparaphenylenes have not been shown to undergo Diels–Alder reactions. Jasti and co-workers incorporated a perylene group into a cycloparaphenylene and reported that its reactivity toward Diels–Alder reactions is similar to that of unstrained perylene.^13^

We recently reported the synthesis and structural properties of cyclic quaterphenylene ethynylene **6**, which adopts a twisted ground state structure bearing ∼19 kcal/mol of strain.^14^ In the molecular structure of compound **6**, the interior bay region of a biphenyl group directly presses against the alkyne. In the present work, we report the heat-mediated intramolecular cyclization of compound **6** to benz[*e*]indeno[1,2,3-*hi*]acephenanthrylene (**7**) as an example of a Diels–Alder-type π-extension reaction of a biphenyl group (Scheme 3).

**Scheme 3:**
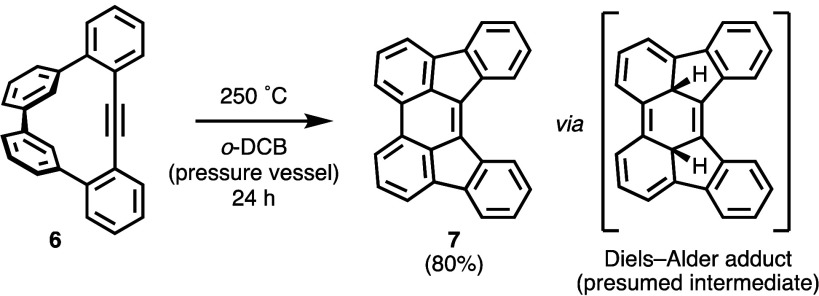
Heat-Mediated Reaction of **6** to **7**

We initially discovered this heat-mediated cyclization reaction by placing ∼5 mg of neat **6** in a vial placed directly on a hot plate heated to roughly ∼270–300 °C for 5 min. The colorless sample of **6** rapidly turned bright yellow, and material partially vaporized and condensed as a solid on the walls of the vial. The ^1^H NMR spectrum of the combined residue showed clean transformation of **6** to **7**, a known compound^15^ whose ^1^H and ^13^C NMR spectra have been reported,^16^ with 87% conversion of **6** based on integration of ^1^H NMR signals (Figure 2b). The reaction was repeated in controlled conditions in solution. Heating **6** in *o*-dichlorobenzene at 250 °C in a pressure vessel for 24 h provided compound **7** with 95% conversion of **6** based on integration of ^1^H NMR signals (Figure 2c) and in 80% isolated yield.

**Figure 2:**
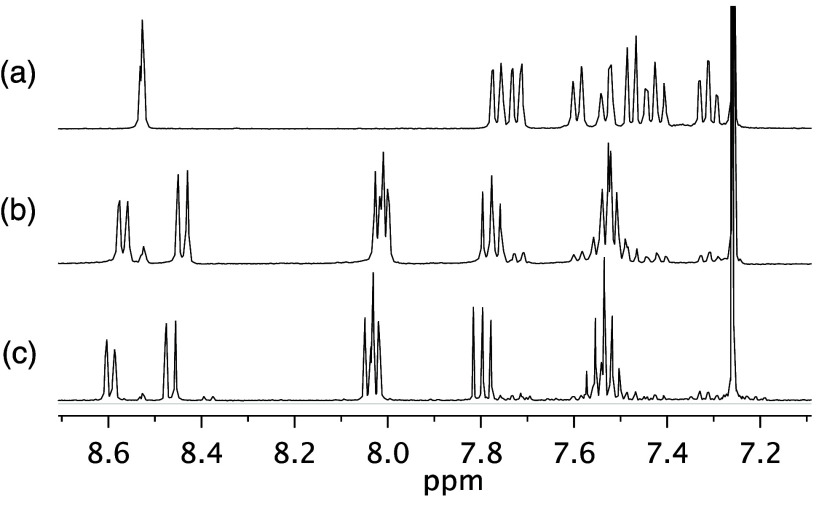
(a) Aromatic region of the ^1^H NMR spectrum of **6**. (b) ^1^H NMR spectrum of material obtained after heating **6** neat at ∼270–300 °C for 5 min, showing 87% conversion to **7**. (c) ^1^H NMR spectrum of material obtained after heating **6** in *o*-DCB for 24 h, showing 95% conversion to **7**. Spectra measured in CDCl_3_ at 400 MHz.

We identified dimethyl derivative **6-Me** as a suitable candidate to study the kinetics of the cyclization reaction by ^1^H NMR and experimentally determine its energetic barrier. Relative integrations of the methyl signals of **6-Me** and **7-Me** were expected (and later confirmed) to be unobscured by other signals and to directly report relative concentrations of the two compounds during the course of the reaction. Compound **6-Me** was prepared from 3-bromo-4-iodotoluene (**8**) in three steps using a strategy similar to our previously reported synthesis of **6** (Scheme 4).^14^ Compound **8** was subjected to the one-pot Sonogashira coupling-deprotection-coupling procedure developed by Brisbois, Grieco, and co-workers,^17^ and the isolated dibromide **9** underwent Suzuki coupling with 3-chlorobenzeneboronic acid to produce dichloride **10**. Ni-mediated Yamamoto coupling conditions accomplished intramolecular homocoupling of the aryl chlorides to yield **6-Me**. As was observed for **6**, Compound **6-Me** undergoes a cyclization reaction at 250 °C over 24 h to generate **7-Me** with >95% conversion (by ^1^H NMR) and in 77% isolated yield.

**Scheme 4:**
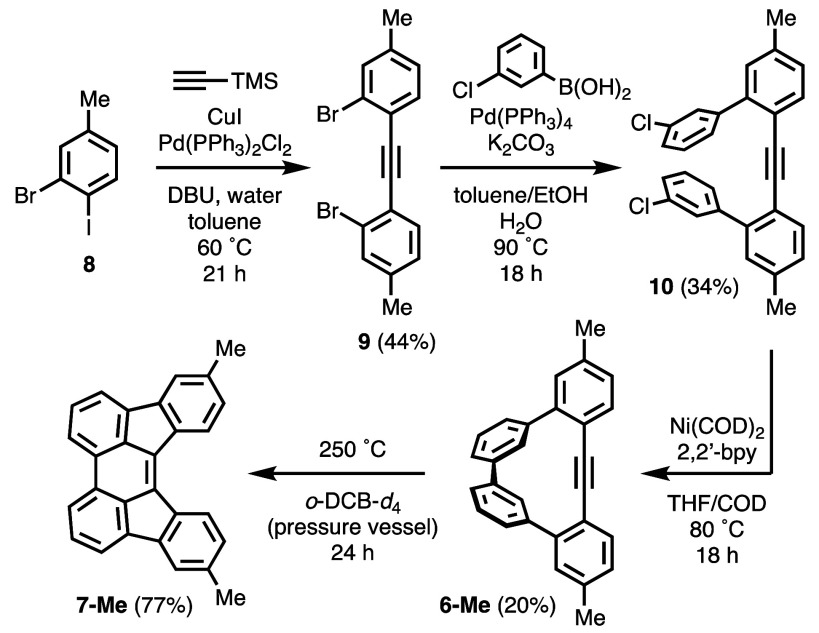
Synthesis of **6**-**Me** and Cyclization to **7**-**Me**

Kinetics studies were performed by heating **6-Me** at 220 °C in *o*-DCB*-d*_4_ (in screw-capped NMR tubes). Three trials were carried out in parallel in one multislot NMR tube heating block. During the reactions, ^1^H NMR measurements of the relative integrations of the methyl signals of **6-Me** and **7-Me** reported the relative amounts of both compounds (Figure 3). The data indicate first-order decomposition of **6-Me**, showing nearly linear plots of −ln([**6-Me**]/[**6-Me**]_0_) versus time. Based on the average value of the three trendlines’ slopes, the rate constant (*k*) of the reaction was found to be ∼1 × 10^–5^ s^–1^. Using the Eyring equation, the Δ*G*^‡^ value for the reaction was determined to be ∼40–41 kcal/mol.^18^

**Figure 3:**
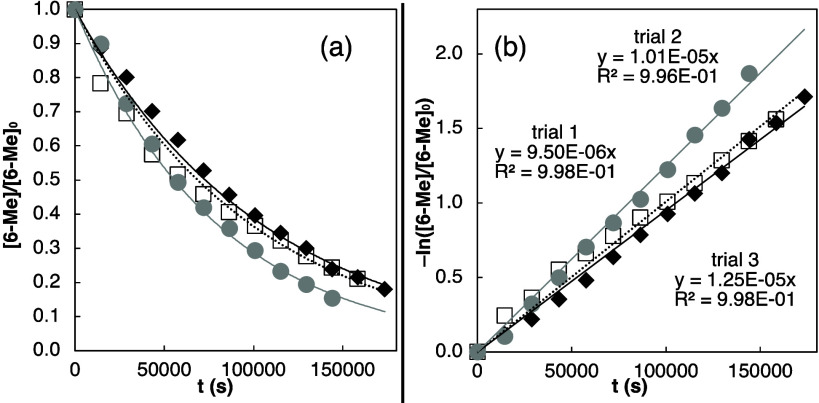
Kinetics data for the reaction of **6-Me** to **7-Me** at 220 °C in *o*-DCB-*d*_4_ (a) Plots of [**6-Me**]/[**6-Me**]_0_ over time for three trials carried out in parallel (trial 1: black solid diamonds and black trendline; trial 2: black empty squares and dashed trendline; trial 3: gray circles and gray trendline). (b) Plots of −ln([**6-Me**]/[**6-Me**]_0_) over time for the three trials.

The mechanism and energetic requirements for the reaction of **6** to **7** were evaluated computationally. DFT calculations using both B3LYP/6-31G(d)^19^ and ωB97X-D/6-311+G(d,p)^20^ methods and basis sets predict that a synchronous, concerted [4 + 2]-cycloaddition followed by concerted loss of H_2_ is a feasible pathway for the reaction (Scheme 5). In this mechanism, the twisted structure of **6** (**6**-*C*_2_) undergoes a ring-flipping conformational change to the higher-energy *C*_*s*_-conformation (**6**-*C*_*s*_) via **TS1**. The *C*_*s*_-conformation permits a suprafacial [4 + 2]-cycloaddition, which occurs via transition state **TS2**, to generate dearomatized intermediate **A**. In the final step, loss of H_2_ generates product **7** via transition state **TS3**. Frey and Krantz’s report of a unimolecular, concerted loss of H_2_ during the decomposition of *cis*-3,6-dimethylcyclohexa-1,4-diene to *p*-xylene,^21^ and Anet’s discovery of suprafacial addition of D_2_ to cyclopentadiene at high pressure and temperature,^22^ support the concerted loss of H_2_ as a feasible process in the final mechanistic step in the transformation of **6** to **7**.

**Scheme 5:**
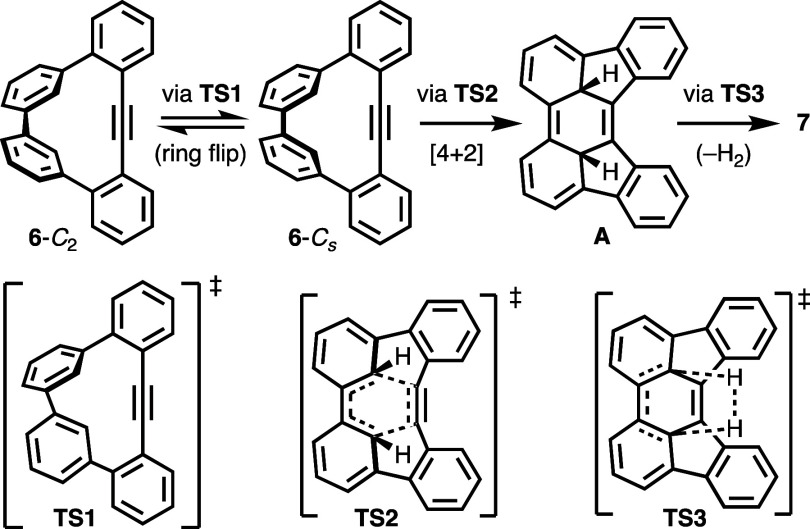
Proposed Mechanism of the Reaction of **6** to **7**

Computed energies (Figure 4) reveal that the barrier to internal rotation from **6**-*C*_2_ to **6**-*C*_*s*_ is only slightly higher than the energy of **6**-*C*_*s*_. The [4 + 2]-cycloaddition that follows requires substantial energy. The calculated *G*_rel_ values for **TS2** (at 220 °C) reasonably match the ∼40–41 kcal/mol value of Δ*G*^‡^ determined from the kinetics experiments with **6-Me** described earlier. Notably, Diels–Alder adduct **A** is lower in energy than **6**-*C*_2_, despite loss of formal aromaticity in the former biphenyl group and the generation of a formally antiaromatic structure. This result is primarily attributable to alleviation of the substantial ring strain that is present in **6**. Concerted loss of H_2_ via **TS3** requires ∼28 kcal/mol from intermediate **A** and provides **7** in a highly exothermic process.

**Figure 4:**
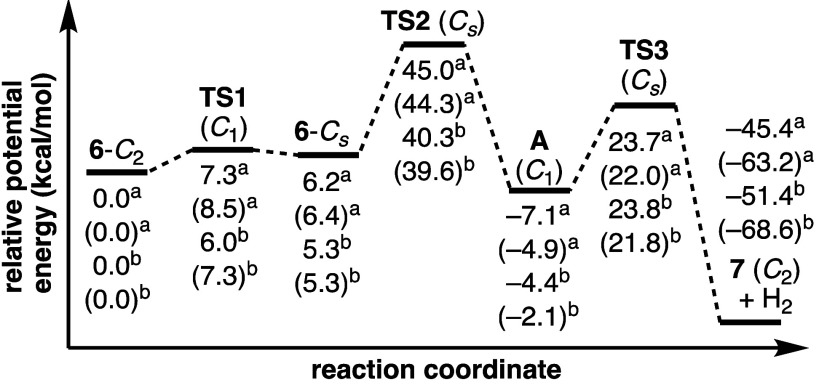
Potential energy diagram of the proposed mechanism for the reaction of **6** to **7**, depicting computed energies of transition states and intermediates in kcal/mol (all energies relative to **6**-*C*_2_). Values not in parentheses denote *E*_rel_, and values in parentheses indicate *G*_rel_ at 220 °C. Values with (^a^) are calculated using ωB97X-D/6-311+G(d,p) and values with (^b^) are calculated using B3LYP/6-31G(d).

Proximity and inward bowing of the alkyne group toward the reacting biphenyl unit, together with relief of considerable strain, likely contribute to compound **6**’s propensity to undergo this cyclization reaction. In computed structure **6**-*C*_*s*_, the distances between the two sets of reacting carbon atoms (biphenyl as diene and alkyne as dienophile) are poised to react at distances of 2.87 Å,^23^ nearer than the sum of the van der Waals radii of two C atoms (∼3.4 Å) (Figure 5). The distances decrease to 2.05 Å in **TS2**, the transition state for the [4 + 2]-cycloaddition, and then continue to shorten to 1.52 Å in Diels–Alder adduct **A**. Bond angles about the alkyne carbon atoms in **6**-*C*_*s*_ distort from linearity (at 172°) and bend inward toward the biphenyl unit. Computational work by Houk, Bickelhaupt, and co-workers demonstrates that cyclononyne, whose alkyne carbons possess bond angles of 168°, is significantly more reactive toward dipolar cycloaddition than a linear alkyne due to the lower relative strain required to reach the reaction’s transition state, a lower HOMO–LUMO gap, and stabilizing orbital interactions.^24^ It is likely that a similar combination of effects operates in the reaction of **6** to **7**.

**Figure 5:**
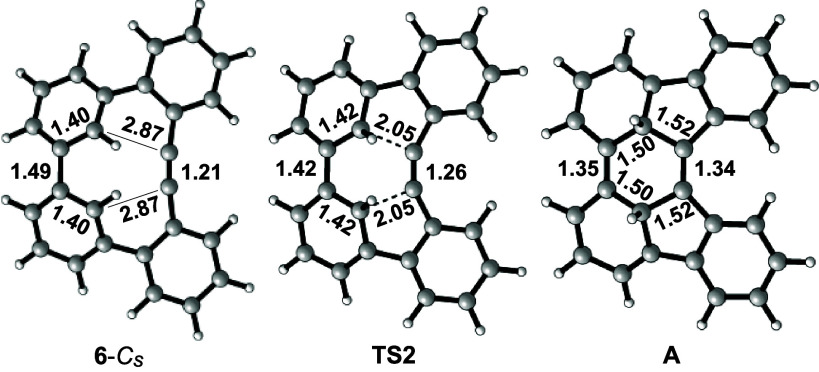
Computed structures of **6**-*C*_*s*_, **TS2**, and **A** (ωB97X-D/6-311+G(d,p)) and selected bond and interatom distances (in Å).

Alternative mechanisms, which are computationally predicted to require much higher energies and, therefore, be less feasible than the one described above, were also considered using B3LYP/6-31G(d) (Figure S1 in Supporting Information). The hypothetical, antarafacial cycloaddition proceeding directly from twisted **6**-*C*_2_ to the *trans*-isomer of **A** was predicted to require 60.6 kcal/mol of activation energy. Asynchronous, diradical pathways were predicted to require 75.7 kcal/mol beginning directly from **6**-*C*_2_ and 74.3 kcal/mol proceeding via **6**-*C*_*s*_.

In conclusion, a biphenyl group in a strained quaterphenylene ethynylene participates in an intramolecular cyclization with a proximal alkyne. This reaction represents an example of a biphenyl derivative undergoing π-extension via a Diels–Alder reaction. Continued efforts to discover other unusual reactivity in strained hydrocarbons are ongoing.

## Experimental Section

### General Remarks

Synthetic procedures were performed under N_2_. Solvents and commercially available reagents were used as received without further purification. THF was purchased as a dry solvent stored under 3 Å molecular sieves. Analytical thin-layer chromatography was performed on Agela Technologies silica gel plates. Melting ranges were recorded on a Vernier Melting Station apparatus. Nuclear magnetic resonance (NMR) spectra were recorded on a Bruker AVANCE II HD spectrometer at 400 MHz (^1^H) or 100 MHz (^13^C). Chemical shifts for ^1^H NMR spectra are reported in ppm relative to TMS (0.00 ppm) or residual solvent signal (5.32 ppm for CHDCl_2_). Chemical shifts for ^13^C{^1^H} (proton-decoupled ^13^C) NMR spectra are reported in ppm relative to TMS (0.00 ppm) or CDCl_3_ (77.0 ppm). Multiplicity is indicated by one or more of the following abbreviations: s (singlet); d (doublet); t (triplet); dd (doublet of doublets); dt (doublet of triplets); dq (doublet of quartets); td (triplet of doublets); ddd (doublet of doublets of doublets); ddq (doublet of doublets of quartets); m (multiplet). Comments such as “dt-like d” indicate that a doublet is present but that each peak of the doublet has the appearance of an unresolved triplet. Coupling constants (*J*) are reported in Hertz (Hz). High-resolution mass spectra (HRMS) were performed at the Mass Spectrometry Laboratory in the School of Chemical Sciences, University of Illinois, Urbana–Champaign; EI-TOF measurements were performed on a Waters GCT Premier mass spectrometer. ASAP-TOF measurements were performed on a Waters Synapt G2-Si mass spectrometer.

### Benz[*e*]indeno[1,2,3-*hi*]acephenanthrylene (**7**)

Compound **6** (29 mg, 0.088 mmol) was dissolved in 1 mL of *o*-DCB in a screw-top pressure vessel. The pressure vessel was closed, and the reaction was heated at 250 °C (high temperature oil bath) for 24 h. A plexiglass blast shield was placed in front of the experiment in case pressure buildup caused the pressure vessel to break. After cooling to room temperature, the solvent was removed *in vacuo* without heating (to obtain the spectrum shown in Figure 2b without causing the reaction to proceed in the process). The resulting yellow, crystalline residue was then triturated in petroleum ether, giving compound **7** as yellow crystals (23 mg, 80%). ^1^H and ^13^C NMR spectral data for compound **7** match those found in the literature.^16^^1^H NMR (400 MHz, CDCl_3_) δ 8.64–8.56 (m, 2H), 8.46 (dd, *J* = 8.1, 0.8 Hz, 2H), 8.16–7.97 (m, 4H), 7.79 (dd, *J* = 8.0, 7.1 Hz, 2H), 7.57–7.50 (m, 4H). ^13^C{^1^H} NMR (100 MHz, CDCl_3_) δ 141.9, 137.8, 137.7, 134.7, 133.1, 128.7, 128.4, 127.8, 127.7, 126.0, 122.0, 121.3, 119.6.

### Bis(2-bromo-4-methylphenyl)ethyne (**9**)

A degassed (sparged with N_2_) mixture of 3-bromo-4-iodotoluene (**8**) (10.00 g, 33.6 mmol), trimethylsilylacetylene (2.4 mL, 1.6 g, 17 mmol, 0.5 equiv), DBU (30.1 mL, 30.8 g, 202 mmol, 6 equiv), H_2_O (0.24 mL, 0.24 g, 14 mmol, 0.4 equiv), and toluene (50 mL) was added to a degassed mixture of Pd(PPh_3_)_2_Cl_2_ (1.42 g, 2.02 mmol, 0.06 equiv) and CuI (0.641 g, 3.37 mmol, 0.10 equiv). The reaction mixture was stirred and heated at 60 °C (oil bath) under N_2_ for 21 h. After cooling the reaction mixture to room temperature, H_2_O and Et_2_O were added. The phases were separated, and the organic phase was washed with 1% HCl (aq) and NaCl (aq), dried over Mg_2_SO_4_, and concentrated. Column chromatography (silica, petroleum ether) afforded compound **9** as a white, crystalline solid (2.75 g, 44%). ^1^H NMR (400 MHz, CDCl_3_) δ 7.69 (d, *J* = 8.1 Hz, 2H), 7.44 (dq, *J* = 2.0 Hz, 0.7 Hz, 2H), 6.79 (ddq, *J* = 8.1, 2.0, 0.7 Hz, 2H) 2.27 (dd, 0.7, 0.7 Hz 6H). ^13^C{^1^H} NMR (100 MHz, CDCl_3_) δ 139.84, 139.81, 133.3, 129.5, 129.4, 96.8, 20.7 (one expected aromatic signal is missing, likely due to signals overlapping). HRMS (EI-TOF) *m*/*z* [M^+^] calcd for C_16_H_12_Br_2_ 361.9306, found 361.9301. mp 107.4–108.1 °C.

### Bis[2-(3-chlorophenyl)-4-methylphenyl]ethyne (**10**)

A 25 mL, three-neck round-bottom flask containing a stir bar was charged with compound **9** (212 mg, 0.582 mmol), 3-chlorobenzeneboronic acid (273 mg, 1.75 mmol, 3 equiv), Pd(PPh_3_)_4_ (67 mg, 0.058 mmol, 0.1 equiv), and K_2_CO_3_ (653 mg, 4.66 mmol, 8 equiv) and evacuated with a vacuum/refilled with N_2_ three times. A degassed (sparged with N_2_) mixture of toluene (8 mL), ethanol (2.7 mL), and H_2_O (1.6 mL) was added to the reaction mixture via cannula. The reaction mixture stirred at 90 °C (oil bath) for 18 h under N_2_. The reaction mixture was cooled to room temperature and added to H_2_O. The mixture was extracted with Et_2_O, and the combined organic phase was dried with MgSO_4_ and concentrated in vacuo. The product was purified by flash column chromatography (petroleum ether as mobile phase) to afford compound **10** (85 mg, 34%) as a cream-colored, crystalline solid. ^1^H NMR (400 MHz, CDCl_3_) δ 7.60 (ddd, *J* = 2.2, 1.7, 0.5 Hz, 2H), 7.41 (dt, *J* = 7.3, 1.5 Hz, 2H), 7.32 (dt-like d, 7.8 Hz, 2H), 7.32–7.28 (m, 2H), 7.26 (apparent td, *J* = 7.7, 0.5 Hz, apparent 2H (central peak appears to overlap with signal from residual CHCl_3_), 7.16 (dt, *J* = 1.8, 0.6 Hz, 2H), 7.11 (ddd, *J* = 7.9, 1.8, 0.7 Hz, 2H), 2.39 (t-like s, 6H). ^1^H NMR (400 MHz, CD_2_Cl_2_) δ 7.61 (t, 1.8 Hz, 2H), 7.43 (ddd, *J* = 7.0, 1.7, 0.8 Hz, 2H), 7.34–7.28 (m, 6H), 7.20 (s, 2H), 7.14 (ddd, *J* = 7.8, 1.7, 0.8 Hz, 2H), 2.39 (s, 6H). ^13^C{^1^H} NMR (100 MHz, CDCl_3_) δ 142.3, 141.7, 138.6, 133.6, 133.0, 130.0, 129.3, 129.0, 128.4, 127.5, 127.4, 118.8, 91.4, 21.4. HRMS (EI-TOF) *m*/*z* [M^+^] calcd for C_28_H_20_Cl_2_ 426.0942, found 426.0935. mp 154.0–156.5 °C.

### 3,16-Dimethyl-19,20-didehydro-5,9:10,14-dimethenodibenzo[*a*,*e*]cyclohexadecene (**6**-Me)

In an N_2_-filled glovebox, a screw-cap pressure vessel was charged with compound **10** (200 mg, 0.468 mmol), 2,2′-bipyridine (292 mg, 1.87 mmol, 4 equiv), and bis(cyclooctadiene) nickel(0) (515 mg, 1.87 mmol, 4 equiv) and a mixture of 1,5-cyclooctadiene (4 mL) and THF (160 mL). The pressure vessel was closed and wrapped in aluminum foil, removed from the glovebox, and heated at 80 °C (oil bath) for 18 h. After cooling the reaction mixture, the solvent was removed *in vacuo* (in a rotary evaporator housed inside and vented to a fume hood to reduce exposure to the strong, unpleasant smell of 1,5-cyclooctadiene). The resulting residue was purified by column chromatography (silica 5% CH_2_Cl_2_/95% petroleum ether) to afford compound **6-Me** as a colorless, crystalline solid (33 mg, 20%). ^1^H NMR (400 MHz, CDCl_3_) δ 8.55 (t, *J* = 1.9 Hz, 2H), 7.62–7.56 (m, 6H), 7.53 (ddd, *J* = 7.8, 2.0, 2H), 7.46 (t, *J* = 7.5 Hz, 2H), 7.12 (ddd, *J* = 7.8, 2H), 2.44 (s, 6H). ^13^C{^1^H} NMR (100 MHz, CDCl_3_) δ 143.6, 140.3, 138.8, 137.9, 136.9, 136.1, 128.6, 128.1, 127.8, 124.8, 123.4, 120.6, 93.7, 21.5. HRMS (EI-TOF) *m*/*z* [M^+^] calcd for C_28_H_20_ 356.1565, found 356.1568. mp 249.0–251.4 °C (sublimed after turning slightly yellow).

### 3,12-Dimethylbenz[*e*]indeno[1,2,3-*hi*]acephenanthrylene (**7**-Me)

A screw-cap pressure vessel was charged with compound **6-Me** (13 mg, 0.037 mmol), which was then dissolved in *o*-DCB-*d*_4_ (1 mL). The pressure vessel was closed and heated to 250 °C (high temperature oil bath) for 24 h. A plexiglass blast shield was placed in front of the experiment in case pressure buildup caused the pressure vessel to break. After cooling the mixture to room temperature, a ^1^H NMR spectrum of the mixture was measured, and integration of the methyl signals of reactant and product showed >95% conversion. Petroleum ether was added to the mixture, causing a yellow, crystalline precipitate to form. The precipitate was collected and washed with petroleum ether to afford compound **7-Me** as yellow crystals (10 mg, 77%). ^1^H NMR (400 MHz, CDCl_3_) δ 8.43 (d, 8.0 Hz, 2H), 8.42 (d, 7.9 Hz, 2H), 7.99 (dd, *J* = 7.1, 0.7 Hz, 2H), 7.83 (s, 2H), 7.76 (dd, *J* = 8.0, 7.1 Hz, 2H), 7.34 (d, 7.9 Hz, 2H), 2.56 (s, 6H). ^13^C{^1^H} NMR (100 MHz, CDCl_3_) δ 142.1, 138.8, 137.7, 135.3, 135.1, 132.5, 128.5, 128.1, 127.6, 125.7, 122.1, 121.9, 119.4, 21.9. HRMS (ASAP-TOF) *m*/*z* [M + H]^+^ calcd for C_28_H_19_ 355.1481, found 355.1477. mp ∼294 °C (partial apparent sublimation and partial decomposition).

### Kinetics Studies

Reactions were carried out on ∼1 mg quantities of **6-Me** in *o*-DCB-*d*_4_ (∼0.5 mL) in Wilmad 5 mm thin wall precision screw cap NMR tubes with solid caps. Compound **7-Me** is sparingly soluble in the solvent and may crystallize out of solution when higher amounts are used (crystallization was not observed in any of the trials reported here but was observed in a prior attempt). The NMR tubes were heated to 220 °C on a Chemglass NMR tube heating block containing 10 slots. The temperature of 220 °C, which is ∼40 °C higher than the boiling point of the solvent, was chosen because it nears the temperature limit of the NMR tubes. All three reactions were carried out simultaneously on the same heating block. After each 4 h period of heating, the tubes were cooled to room temperature (pausing the reaction), and ^1^H NMR spectra were recorded at room temperature. The value of [**6-Me**]/[**6-Me**]_0_ in each measurement was determined by dividing the integration of the methyl signal of **6-Me** by the sum of integrations of the methyl signals of **6-Me** and **7-Me** with the assumption that no side reactions were occurring (an assumption that was confirmed during the experiment). Measurements were recorded until the amount of **6-Me** reached ∼20% of its original amount. As the reactions proceeded, the baseline of the spectra became distorted due to very broad signals from a polymeric impurity deriving from the screw cap. A sample containing only *o*-DCB-*d*_4_ was heated and inverted multiple times to allow the solvent to contact the screw cap, and the resulting spectrum showed the same broad signals noted in the kinetics experiments. A baseline correction using a Whittaker smoother^25^ was applied to mitigate the effects of this baseline distortion and give reliable integrations for the methyl signals.

## Computational Methods

Calculations were performed using GAMESS version R1 released in 2020.^26^ All gas-phase geometries were optimized using the density functional B3LYP^19^ and the 6-31G(d) basis set *in vacuo*. Then gas-phase geometries were optimized using the density functional ωB97X-D^20^ and the 6-311+G(d,p) basis set *in vacuo*. Geometry optimizations and transition state optimizations were performed with tight convergence criteria of 10^–5^ hartree/bohr. Vibrational frequency calculations confirmed that the ground state geometries were minima, and the transition state geometries were saddle points. Using Truhlar’s quasiharmonic correction,^27^ free energies were calculated at 493.15 K and 1 atm. Geometries are displayed using CYLview.^28^ All calculations were first performed without symmetry constraints. Optimized structures that were found to be very nearly symmetric were optimized a second time in the point group determined by the initial calculation and are labeled by the notation “(true [point group])” in the Supporting Information.

## Data Availability

The data underlying this study are available in the published article and in its Supporting Information.

## Supporting Information Available

^1^H NMR and ^13^C NMR spectra of all prepared compounds, data from kinetics studies, and computational details of all calculated structures (PDF)
Models of all calculated structures (XYZ)

## Supplementary Material

jo2c02280_si_001.pdf

jo2c02280_si_002.xyz
