# Supplementary material for: Intramolecular Diels–Alder Reaction of a Biphenyl Group in a Strained meta-Quaterphenylene Acetylene
Source: J Org Chem. 2023 Jan 26;89(13):9620–6. doi: 10.1021/acs.joc.2c02280 (PMC11232012; doi:10.1021/acs.joc.2c02280)
Supplement: Supplementary file 1 — jo2c02280_si_001.pdf [file jo2c02280_si_001.pdf]

Supporting Information for
Intramolecular Diels–Alder Reaction of a Biphenyl Group in a Strained meta-*Quaterphenylene Acetylene*

Komal Mittal, Ashley V. Pham, Amanda G. Davis, Abigail D. Richardson, Clement De Hoe, Ryan T. Dean, Vi Baird, Ashley Ringer McDonald, and Derik K. Frantz*

Department of Chemistry and Biochemistry, California Polytechnic State University, 1 Grand Avenue, San Luis Obispo, CA 93407

dfrantz@calpoly.edu

Contents

1. ^{1}H-NMR and ^{13}C{^{1}H}-NMR Spectra of Prepared Compounds S2
1.1. ^{1}H-NMR Spectrum of Compound **7** S2
1.2. ^{13}C{^{1}H}-NMR Spectrum of Compound **7** S3
1.3. ^{1}H-NMR Spectrum of Compound **9** S4
1.4. ^{13}C{^{1}H}-NMR Spectrum of Compound **9** S5
1.5. ^{1}H-NMR Spectrum of Compound **10** (in $CDCl_3$) S6
1.6. ^{1}H-NMR Spectrum of Compound **10** (in CD_2Cl_2) S7
1.7. ^{13}C{^{1}H}-NMR Spectrum of Compound **10** S8
1.8. ^{1}H-NMR Spectrum of Compound **6-Me** S9
1.9. ^{13}C{^{1}H}-NMR Spectrum of Compound **6-Me** S10
1.10. ^{1}H-NMR Spectrum of Compound **7-Me** S11
1.11. ^{13}C{^{1}H}-NMR Spectrum of Compound **7-Me** S12

2. Experimental Data for Kinetics Studies S13

3. Computed Structures S16
3.1. B3LYP/6-31G(d) Coordinates and Thermochemistry Data S16
3.2. ωB97X-D/6-311+G(d,p) Coordinates and Thermochemistry Data S28
3.3. Alternative Mechanisms S40
3.3.1. Coordinates and Thermochemistry Data for Alternative Mechanisms S41

1. ^{1}H-NMR and ^{13}C{^{1}H}-NMR Spectra of Prepared Compounds

1.1. ^{1}H-NMR Spectrum of Compound **7** (400 MHz, $CDCl_3$)

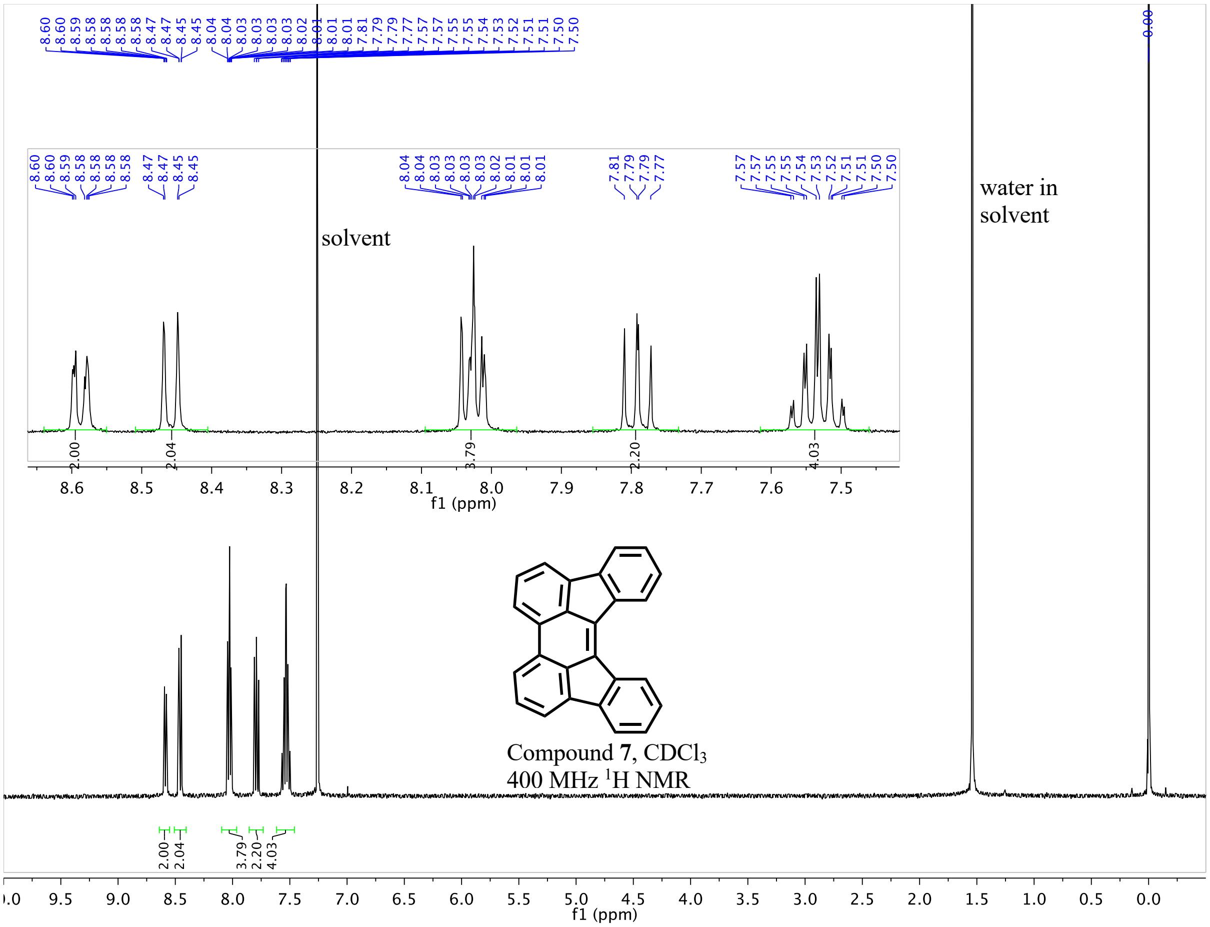

1.2. $^{13}C\{^1H\}$-NMR Spectrum of Compound **7** (100 MHz, $CDCl_3$)

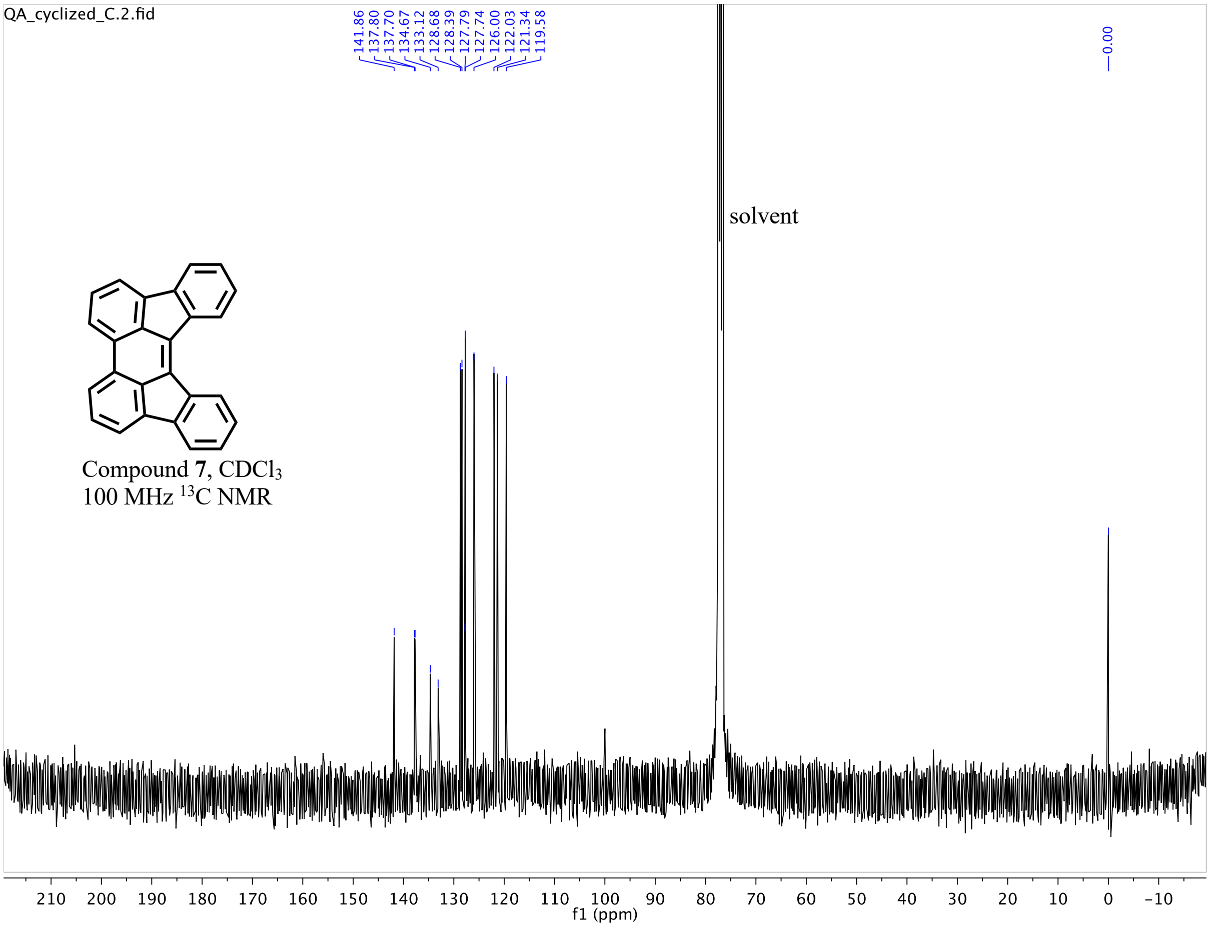

1.3. ^{1}H-NMR Spectrum of Compound **9** (400 MHz, $CDCl_3$)

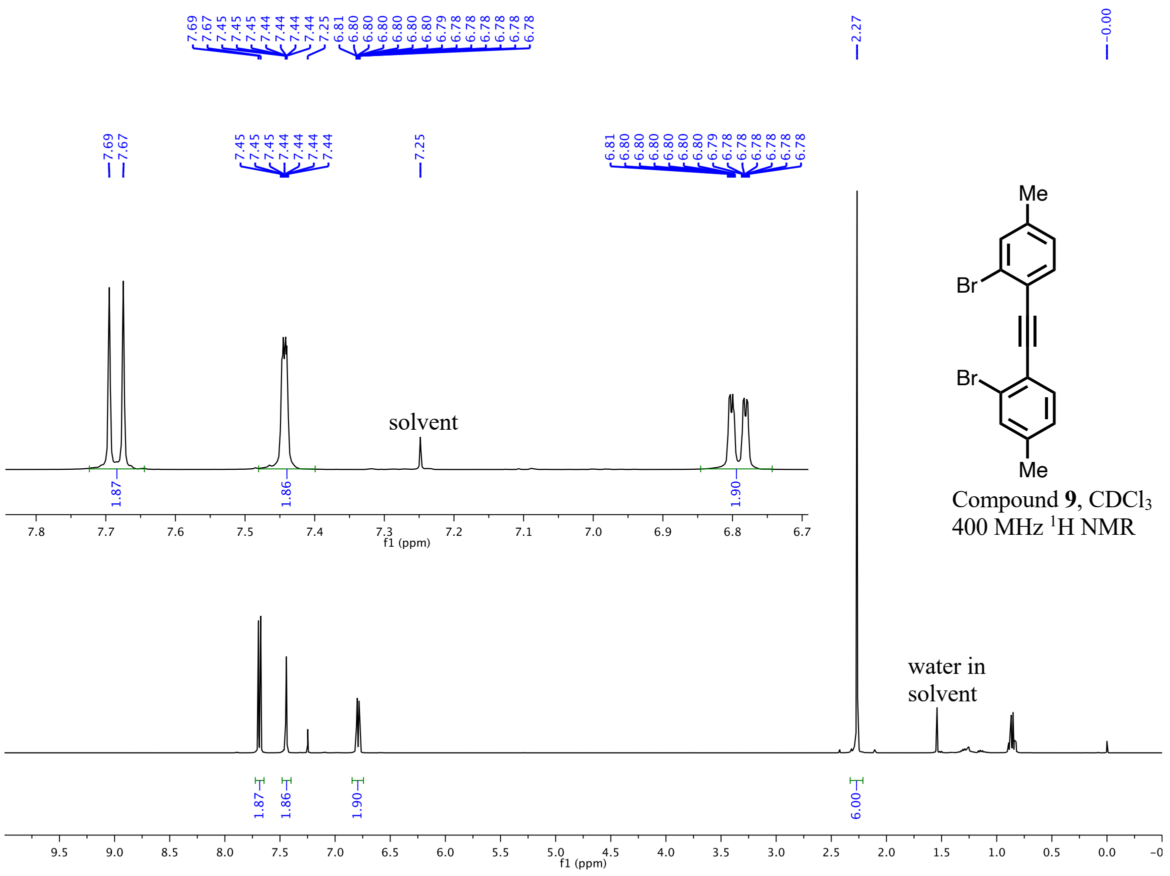

1.4. $^{13}C\{^{1}H\}$-NMR Spectrum of compound **9** (100 MHz, $CDCl_3$)

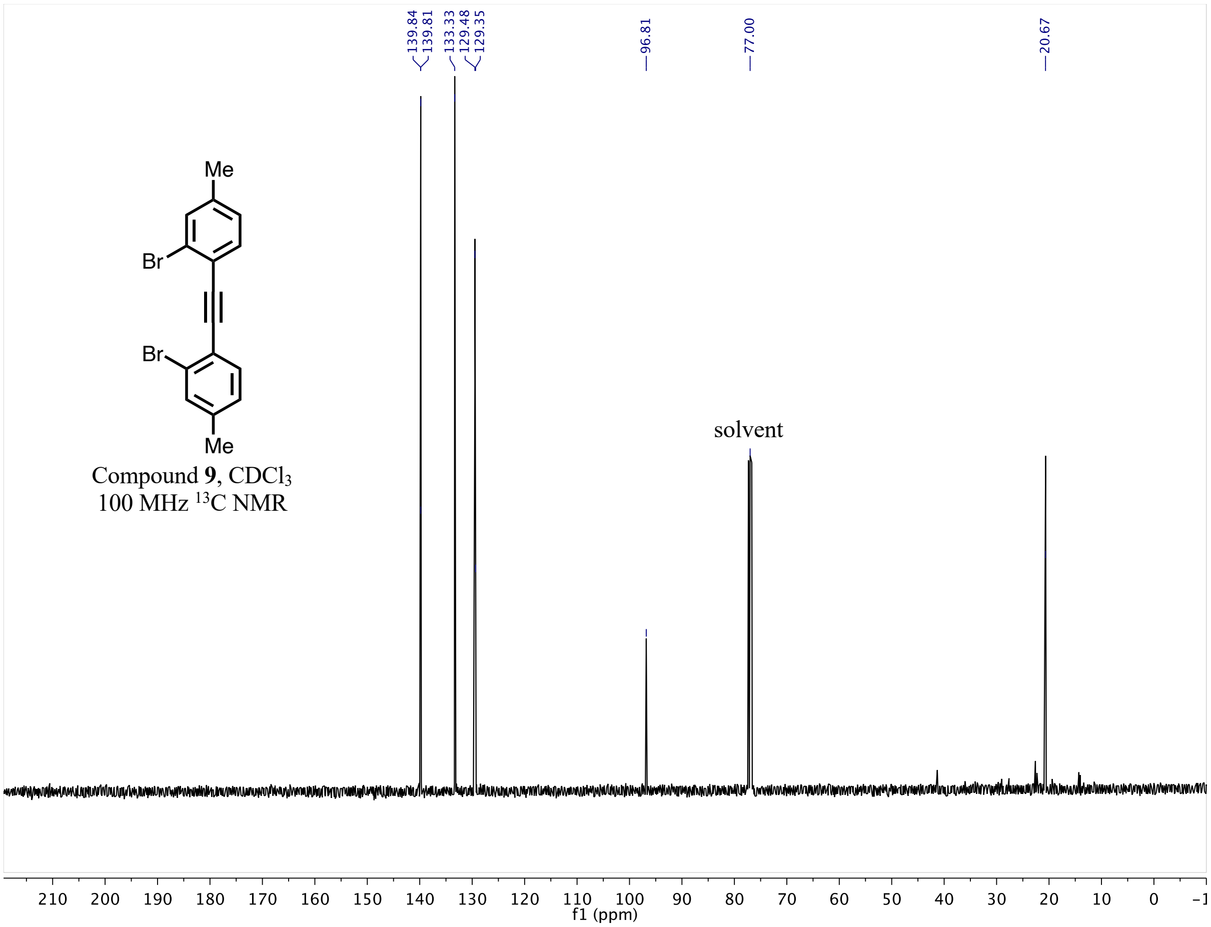

1.5. ^{1}H-NMR Spectrum of compound **10** (400 MHz, $CDCl_3$)

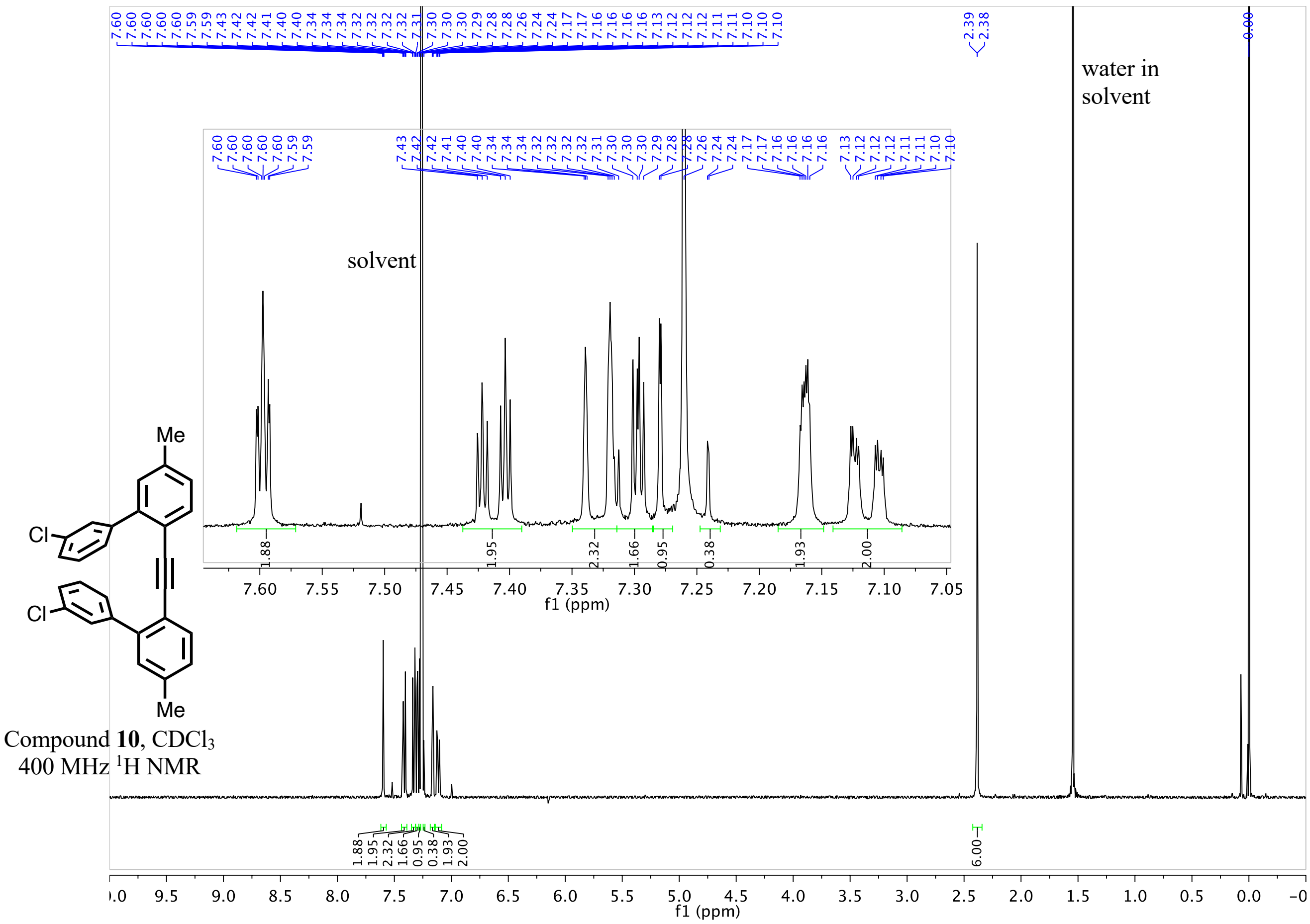

1.6. ^{1}H-NMR Spectrum of Compound **10** (400 MHz, CD_2Cl_2)

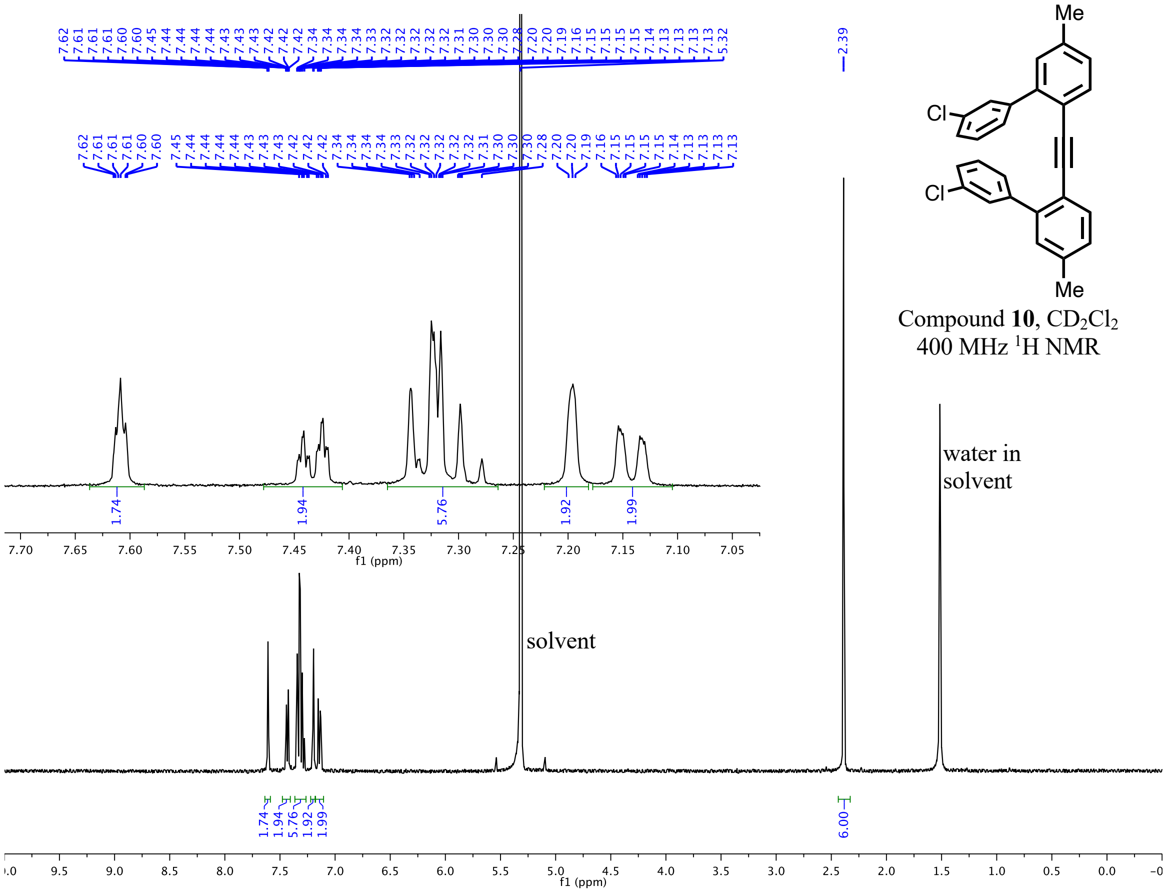

1.7. $^{13}C\{^{1}H\}$-NMR Spectrum of Compound **10** (100 MHz, $CDCl_3$)

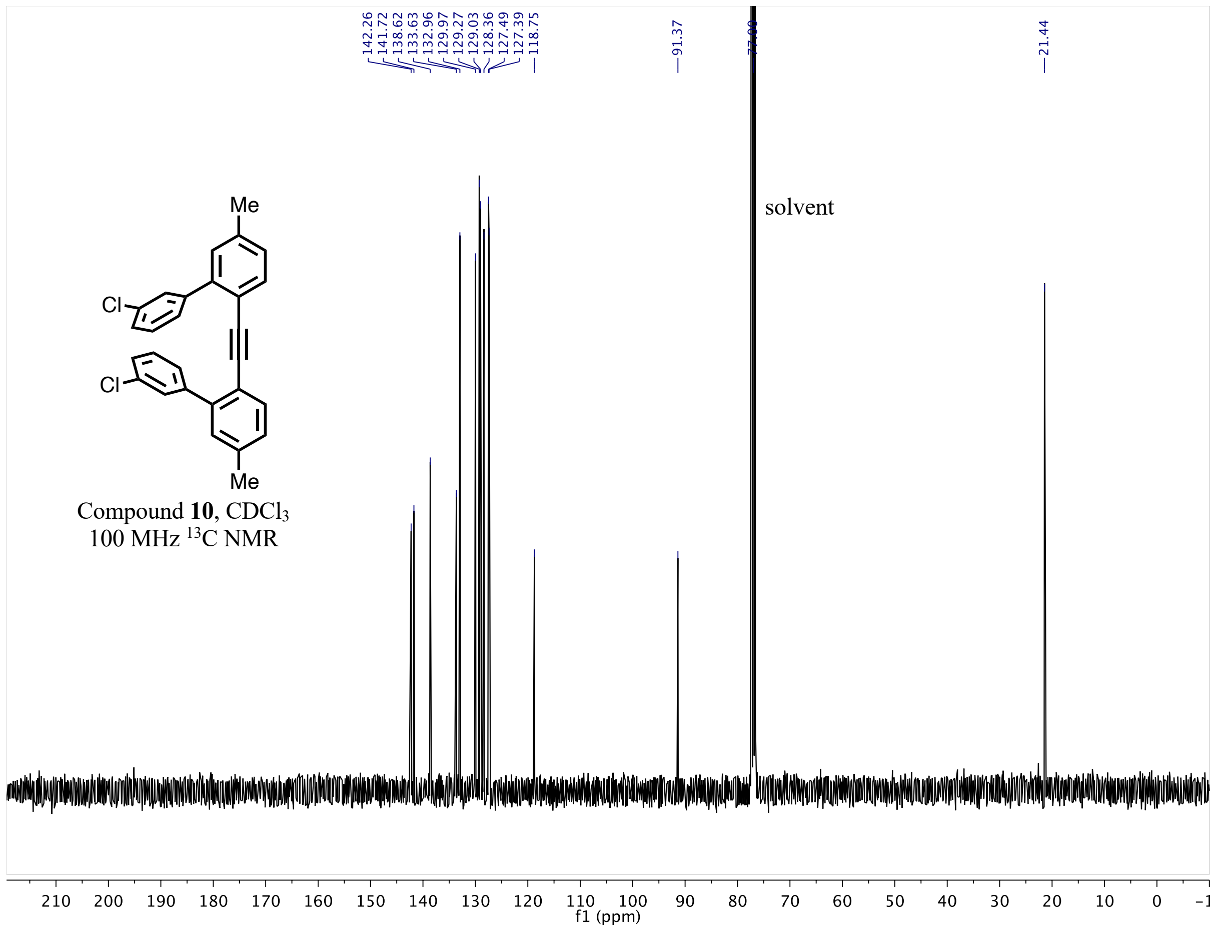

1.8. ^{1}H-NMR Spectrum of Compound **6-Me** (400 MHz, $CDCl_3$)

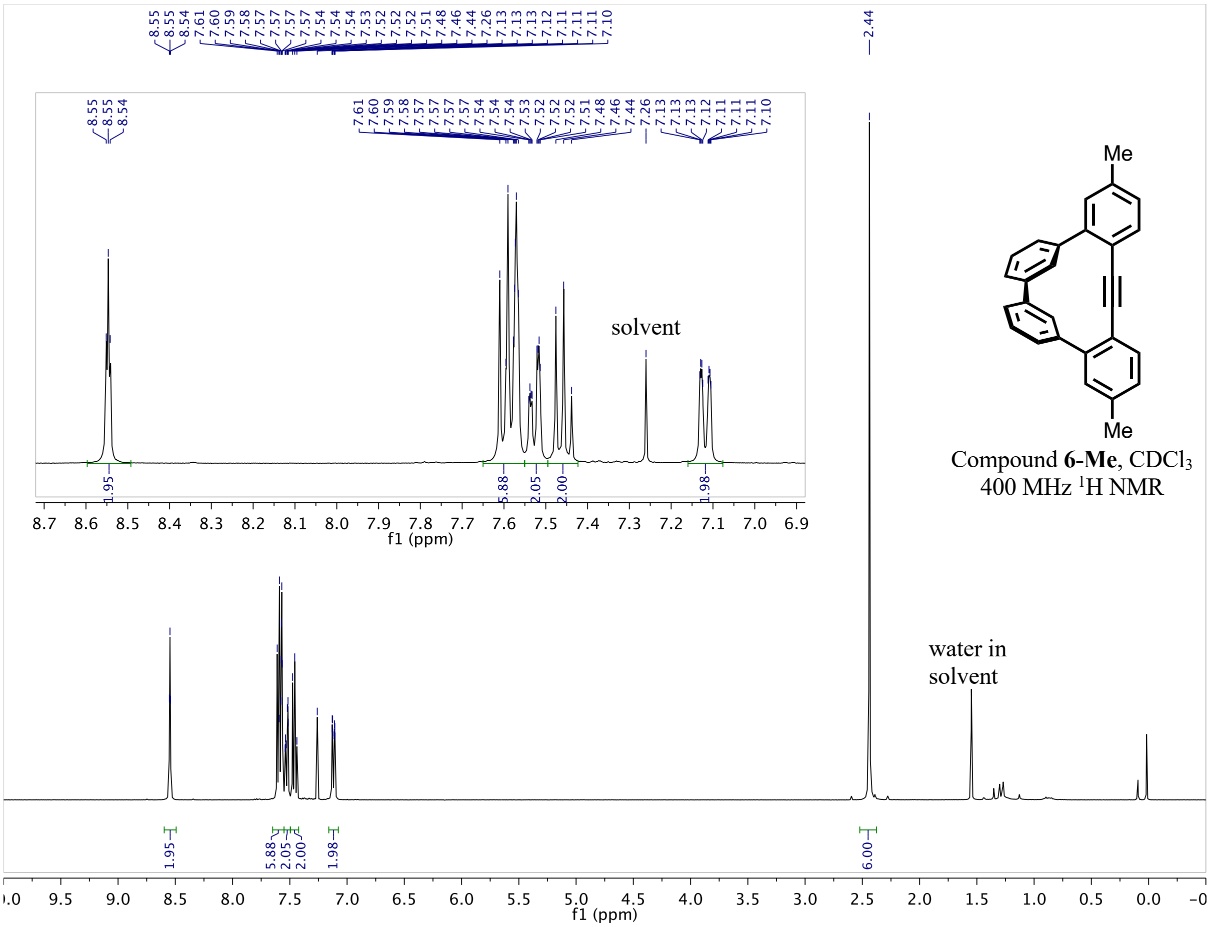

1.9. $^{13}C\{^{1}H\}$-NMR Spectrum of Compound **6-Me** (100 MHz, $CDCl_3$)

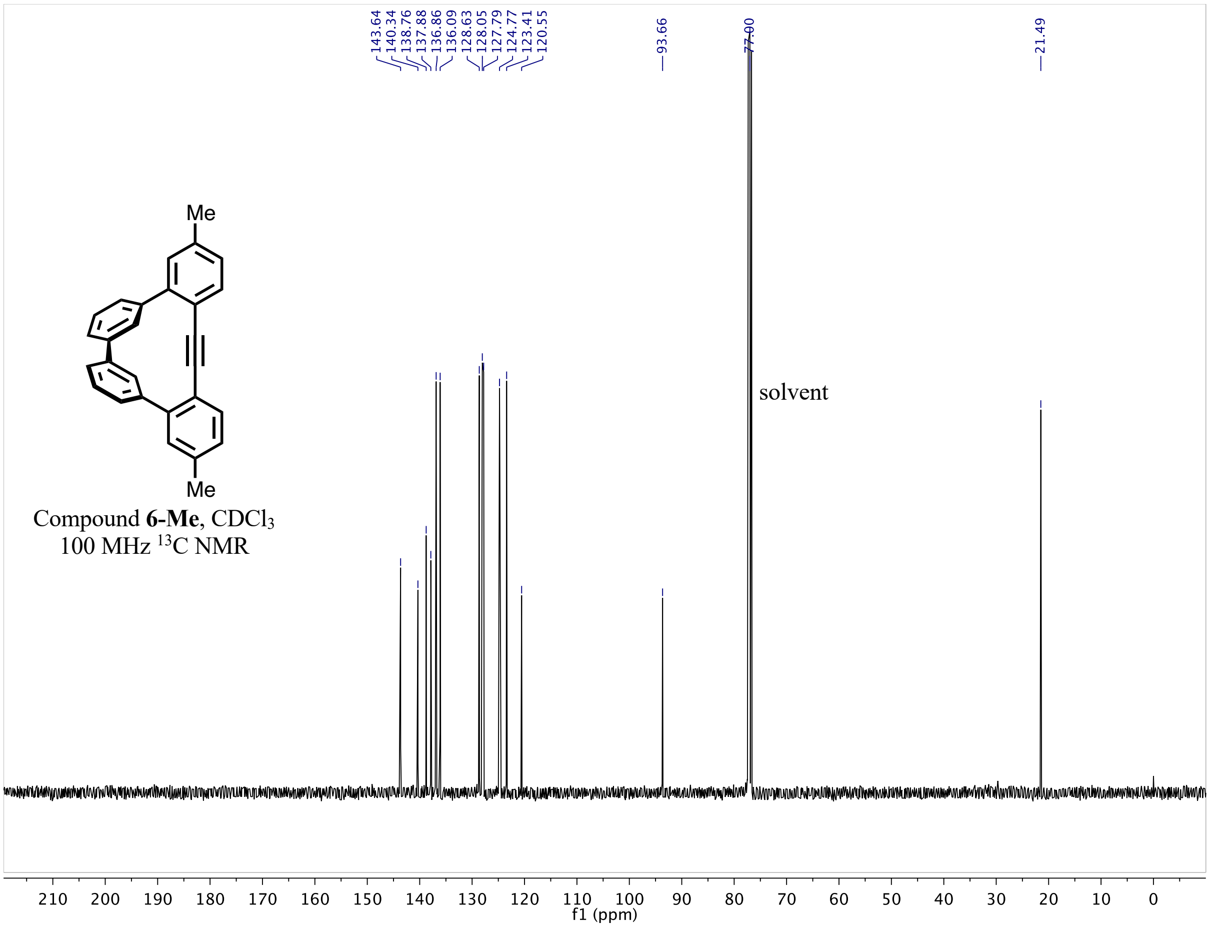

1.10. ^{1}H-NMR Spectrum of Compound **7-Me** (400 MHz, $CDCl_3$)

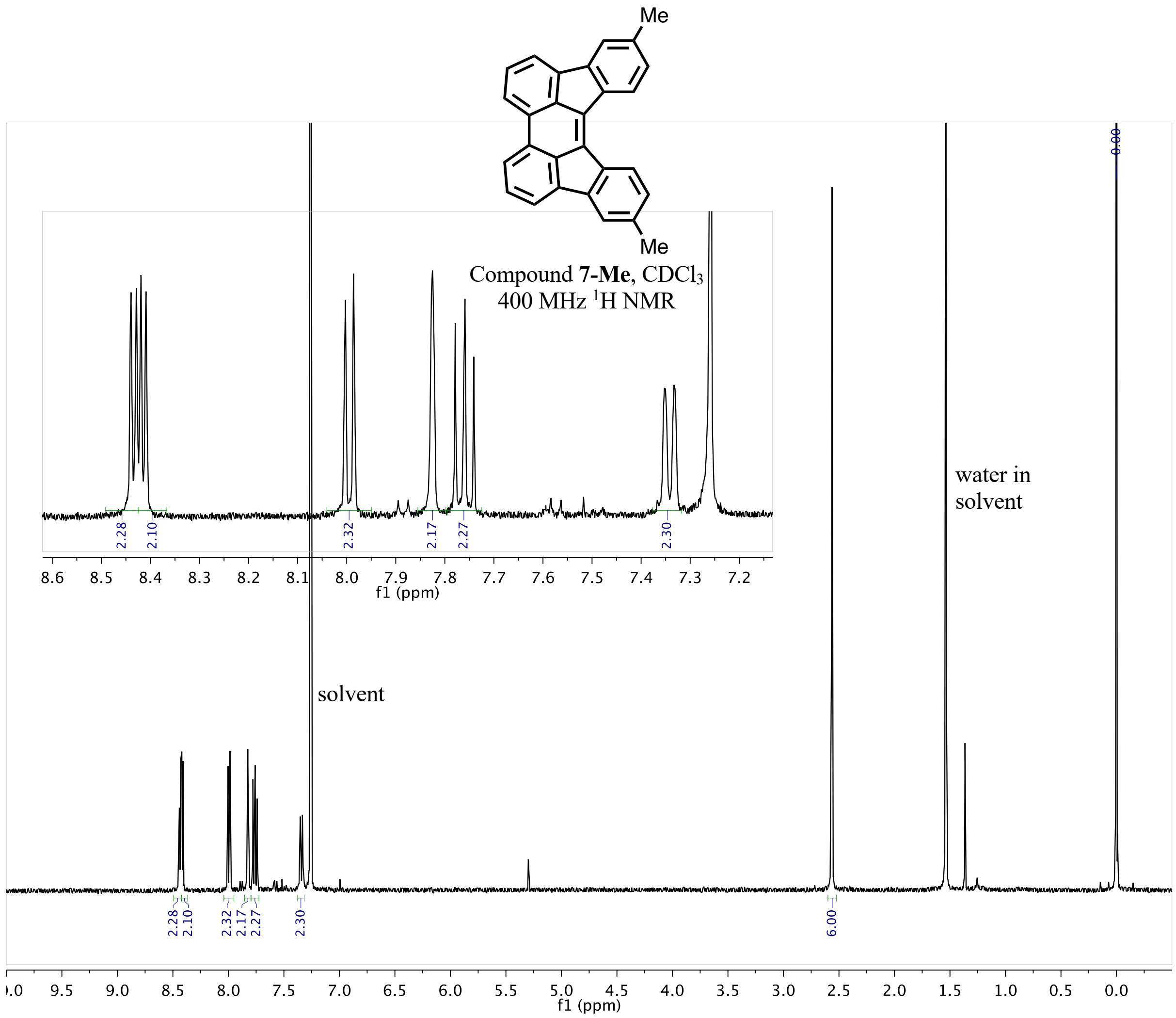

1.11. $^{13}C\{^{1}H\}$-NMR Spectrum of Compound **7-Me** (100 MHz, $CDCl_3$)

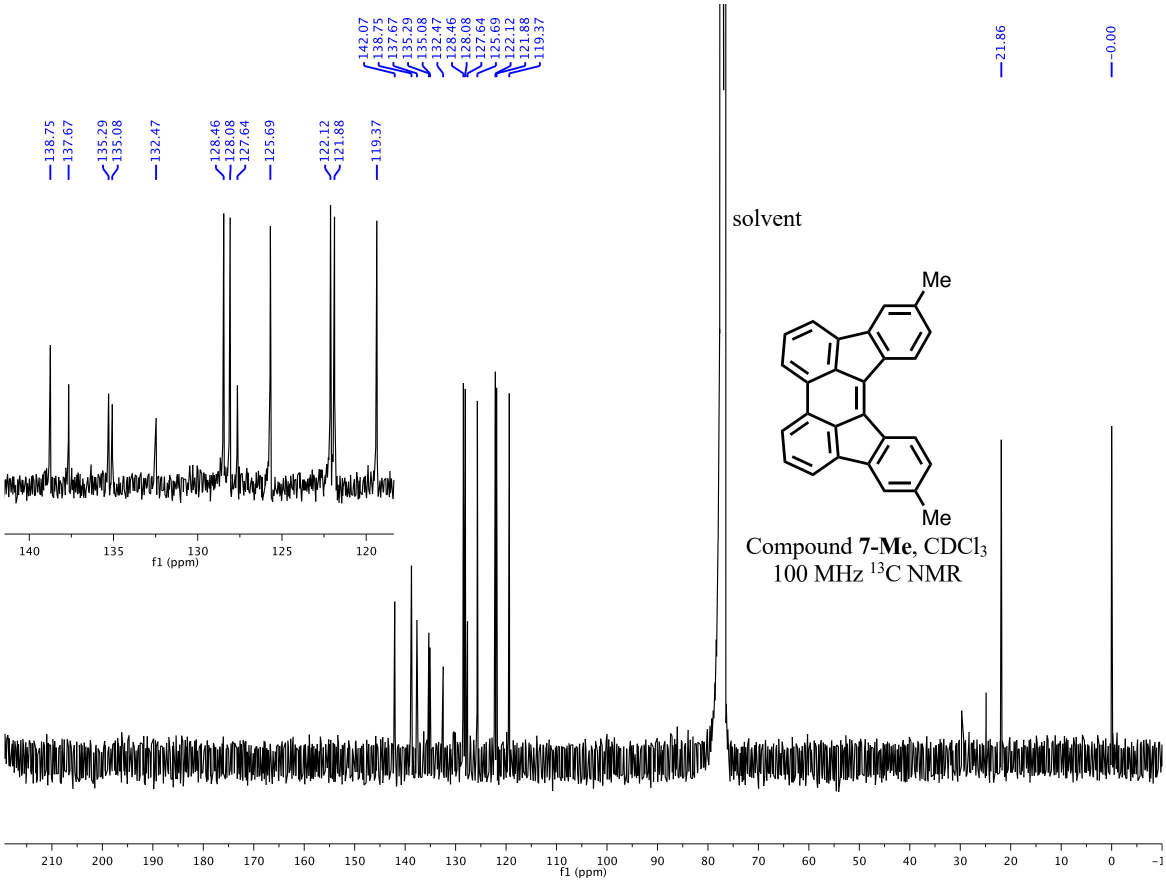

2. Experimental Details for Kinetics Studies

Data for Trial 1

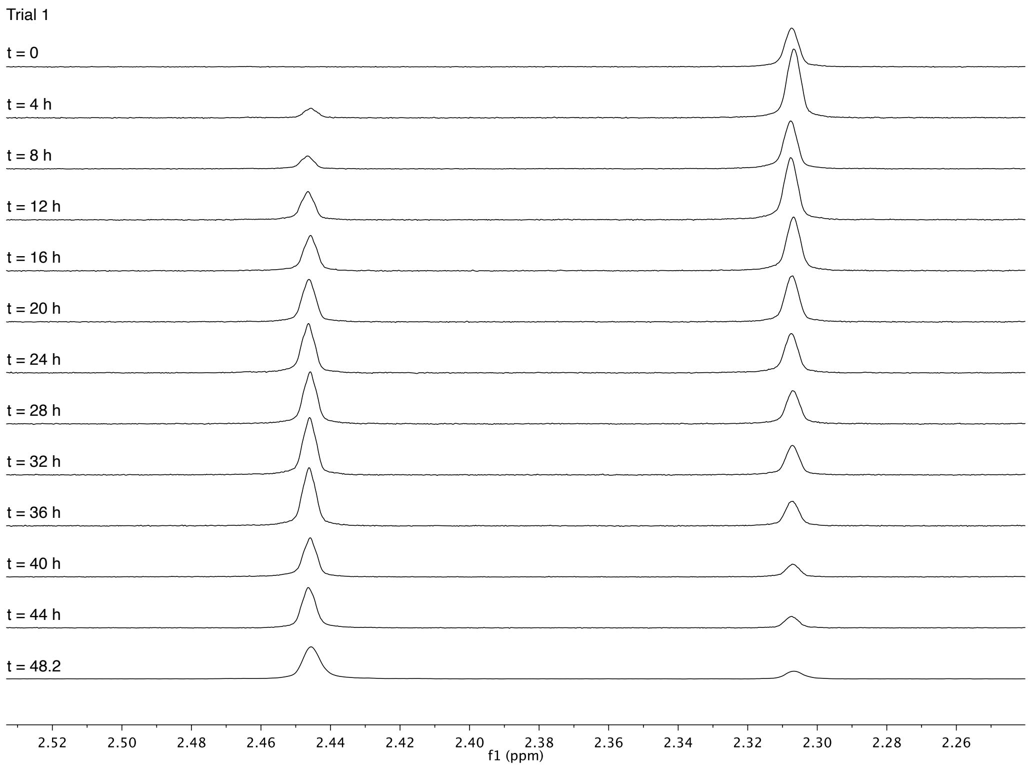

Trial 1

Time (h)	Time (s)	Int [R][a]	Int [P]	[R]/[R]o	–ln([R]/[R]o)
0	0	1.000	0.000	1	0
4	14400	1.000	0.128	0.887	0.120
8	28800	1.000	0.247	0.802	0.221
12	43200	1.000	0.425	0.702	0.354
16	57600	1.000	0.619	0.618	0.482
20	72000	1.000	0.892	0.529	0.638
24	86400	1.000	1.192	0.456	0.785
28	100800	1.000	1.523	0.396	0.925
32	115200	1.000	1.899	0.345	1.064
36	129600	1.000	2.325	0.301	1.201
40	144000	1.000	3.171	0.240	1.428
44	158400	1.000	3.650	0.215	1.537
48.2	173520	1.000	4.547	0.180	1.713

[a] Int [R] is integration of methyl signal of **6-Me** and is normalized to 1.00 for all measurements. Int [P] is integration of methyl signal of **7-Me** relative to Int [R].

Data for Trial 2

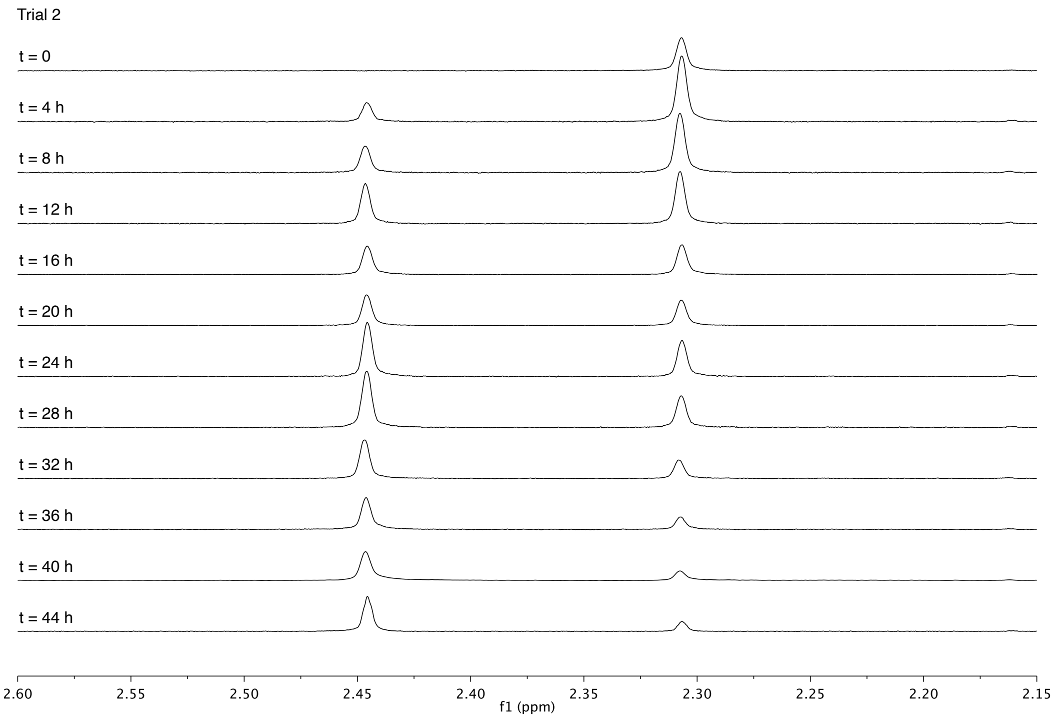

Trial 2

Time (h)	Time (s)	Int [R]	Int [P]	[R]/[R]o	–ln([R]/[R]o)
0	0	1.000	0.000	1	0
4	14400	1.000	0.276	0.784	0.244
8	28800	1.000	0.438	0.695	0.363
12	43200	1.000	0.738	0.575	0.553
16	57600	1.000	0.937	0.516	0.661
20	72000	1.000	1.178	0.459	0.778
24	86400	1.000	1.463	0.406	0.901
28	100800	1.000	1.738	0.365	1.007
32	115200	1.000	2.102	0.322	1.132
36	129600	1.000	2.610	0.277	1.284
40	144000	1.000	3.112	0.243	1.414
44	158400	1.000	3.762	0.210	1.561

[a] Int [R] is integration of methyl signal of **6-Me** and is normalized to 1.00 for all measurements. Int [P] is integration of methyl signal of **7-Me** relative to Int [R].

Data for Trial 3

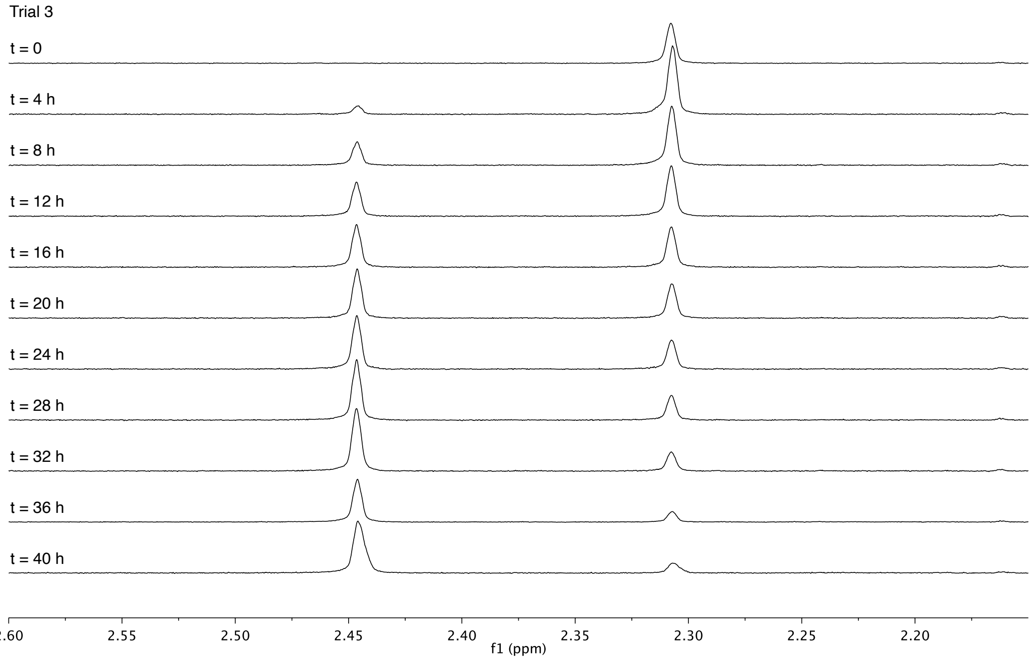

Trial 3

Time (h)	Time (s)	Int [R]	Int [P]	[R]/[R]o	–ln([R]/[R]o)
0	0	1.000	0.000	1	0
4	14400	1.000	0.112	0.899	0.106
8	28800	1.000	0.380	0.725	0.322
12	43200	1.000	0.649	0.606	0.500
16	57600	1.000	1.023	0.494	0.705
20	72000	1.000	1.381	0.420	0.868
24	86400	1.000	1.786	0.359	1.025
28	100800	1.000	2.402	0.294	1.224
32	115200	1.000	3.287	0.233	1.456
36	129600	1.000	4.140	0.195	1.637
40	144000	1.000	5.481	0.154	1.869

[a] Int [R] is integration of methyl signal of **6-Me** and is normalized to 1.00 for all measurements. Int [P] is integration of methyl signal of **7-Me** relative to Int [R].

3. Computed Structures

3.1. B3LYP/6-31G(d) Coordinates and Thermochemistry Data

6-C_2
42 atoms
Zero imaginary vibrational frequencies

C	0.00000000	0.00000000	0.00000000
C	-0.45839911	-1.28341281	0.33050637
C	0.36988403	-2.13144320	1.07013948
C	1.60203624	-1.63108557	1.52020000
C	2.13037512	-0.41556284	1.09835541
C	1.29023194	0.41837243	0.33457721
H	1.65487553	1.37340858	-0.03459730
C	3.55357313	-0.10750583	1.40041876
C	4.57027622	-1.11821118	1.44945260
C	4.36953452	-2.52386249	1.21849698
C	4.29189145	-3.73402558	1.06762045
C	4.31016221	-5.15396894	0.83781736
C	3.17226675	-6.02566060	0.88835170
C	1.80125467	-5.54040491	1.19764240
C	0.85755660	-6.27074592	1.94634345
C	-0.36799520	-5.69322974	2.28750847
C	-0.65690980	-4.35582787	1.97901367
C	0.27618007	-3.61074865	1.25388819
C	1.43481857	-4.25933611	0.79834469
H	2.11177019	-3.69568564	0.17298382
H	-1.56633460	-3.89332849	2.35460101
H	-1.08350849	-6.27253087	2.86592617
H	1.09311284	-7.27156847	2.29894193
C	3.37965913	-7.39397261	0.65450483
C	4.64009240	-7.92219756	0.38708967
C	5.74801074	-7.07483546	0.34242813
C	5.57749913	-5.71290436	0.56244733
H	6.43380627	-5.04586287	0.53060445
H	6.73877965	-7.46965675	0.13425801
H	4.75353814	-8.98798522	0.20709191
H	2.51764093	-8.05513079	0.66514768
C	5.89887424	-0.72668148	1.72562215
C	6.24252283	0.60165449	1.94796510
C	5.25281012	1.58447815	1.90230305
C	3.93518914	1.22274620	1.63308652
H	3.16555432	1.98954726	1.62265726
H	5.50234392	2.62673701	2.08301361
H	7.27536588	0.86626158	2.15803599
H	6.66241403	-1.49824467	1.75694436
H	2.20194342	-2.26683665	2.15555609
H	-1.41899063	-1.63235317	-0.04047391
H	-0.63408128	0.65644194	-0.59088312

6-C_2 (true *C_2*)
42 atoms
Zero imaginary vibrational frequencies

C	0.00000000	0.00000000	0.00000000
C	5.59922940	2.54527785	0.00000000
C	1.29701937	0.37744207	-0.37830576
C	4.30221004	2.16783578	-0.37830576
C	2.06294927	1.14799718	0.50018379
C	3.53628013	1.39728067	0.50018379
C	1.46920190	1.57741013	1.69793972
C	4.13002751	0.96786772	1.69793972
C	0.23882164	1.11200968	2.14986836
C	5.36040777	1.43326817	2.14986836
C	-0.51137970	0.31834481	1.26015025
C	6.11060910	2.22693303	1.26015025
H	-1.47398965	-0.08480821	1.56413514
H	7.07321905	2.63008606	1.56413514
C	-0.17228917	1.40211902	3.54910092
C	5.77151857	1.14315883	3.54910092
C	0.76665921	1.49270214	4.62919885
C	4.83257019	1.05257571	4.62919885
C	2.19069780	1.32269018	4.51906644
C	3.40853160	1.22258767	4.51906644
H	3.55641364	0.31001072	2.33481211
H	2.04281577	2.23526713	2.33481211
H	3.88109783	2.52664335	-1.31416513
H	1.71813158	0.01863450	-1.31416513
H	6.19263524	3.15961864	-0.67275353
H	-0.59340584	-0.61434079	-0.67275353
C	7.13260304	0.96839662	3.84260598
C	-1.53337364	1.57688123	3.84260598
C	7.59090626	0.71883312	5.13382529
C	-1.99167685	1.82644472	5.13382529
C	6.67772679	0.63389279	6.18556764
C	-1.07849738	1.91138506	6.18556764
C	5.32122869	0.79723239	5.92955638
C	0.27800071	1.74804545	5.92955638
H	4.60318739	0.73457332	6.74180504
H	0.99604201	1.81070453	6.74180504
H	7.01789466	0.43887065	7.19906161
H	-1.41866526	2.10640720	7.19906161
H	8.65417296	0.58409240	5.31415267
H	-3.05494355	1.96118545	5.31415267
H	7.84651737	1.01053893	3.02468854
H	-2.24728797	1.53473892	3.02468854

6-*C_s*
42 atoms
Zero imaginary vibrational frequencies

C	0.00000000	0.00000000	0.00000000
C	-0.30082166	-1.31130634	-0.38645240
C	0.71094653	-2.14794433	-0.88241780
C	1.97519512	-1.56728151	-1.06769541
C	2.33687396	-0.33367538	-0.53828131
C	1.31248422	0.47534071	-0.01175894
H	1.54113965	1.43975964	0.43322202
C	3.78385464	-0.00856851	-0.43684407
C	4.78565248	-1.01517226	-0.22536086
C	4.56362396	-2.43631459	-0.15753014
C	4.51264089	-3.65915771	-0.15847122
C	4.62174356	-5.09309812	-0.22433195
C	3.54293225	-6.01800588	-0.42817453
C	2.12659661	-5.57722558	-0.52025018
C	1.05010331	-6.28842740	0.04212410
C	-0.21778258	-5.70529707	0.07239422
C	-0.41446860	-4.38031469	-0.33315620
C	0.65130862	-3.64123877	-0.86972393
C	1.85854266	-4.32877647	-1.07106086
H	2.67627699	-3.84410212	-1.57459377
H	-1.38120483	-3.91423998	-0.16334446
H	-1.05057023	-6.25969234	0.49800786
H	1.20897790	-7.26074119	0.50030304
C	3.84899405	-7.38681934	-0.48672203
C	5.14828581	-7.86699896	-0.34874864
C	6.19701017	-6.96934143	-0.14203207
C	5.93081052	-5.60676064	-0.08434911
H	6.74129026	-4.90197053	0.07560757
H	7.21706354	-7.32661108	-0.02937460
H	5.34085689	-8.93502434	-0.40865458
H	3.03950671	-8.08920895	-0.66574993
C	6.13101601	-0.60685441	-0.08430671
C	6.50462074	0.72998848	-0.15005355
C	5.53080151	1.70707857	-0.36404212
C	4.19744730	1.33103410	-0.50150238
H	3.44635990	2.09393456	-0.68627207
H	5.80706512	2.75605151	-0.43006877
H	7.54982791	1.00549617	-0.03854547
H	6.88241002	-1.37312687	0.08108225
H	2.76023499	-2.13093345	-1.53808797
H	-1.30874337	-1.68825212	-0.23654035
H	-0.79223424	0.63110351	0.39519048

6-C_s (true C_s)
42 atoms
Zero imaginary vibrational frequencies

C	0.00000000	0.00000000	0.00000000
C	0.00000000	0.00000000	5.70750402
C	-0.25986592	0.38966684	1.31885117

C	-0.25986592	0.38966684	4.38865286
C	0.76479174	0.93730860	2.10639851
C	0.76479174	0.93730860	3.60110551
C	1.99528000	1.16648659	1.47103604
C	1.99528000	1.16648659	4.23646799
C	2.32531405	0.63462610	0.22912655
C	2.32531405	0.63462610	5.47837747
C	1.28999704	0.05840284	-0.53036085
C	1.28999704	0.05840284	6.23786487
H	1.49656075	-0.38915379	-1.49857655
H	1.49656075	-0.38915379	7.20608058
C	3.76035435	0.57958731	-0.15427225
C	3.76035435	0.57958731	5.86177628
C	4.80556618	0.40270209	0.81363840
C	4.80556618	0.40270209	4.89386562
C	4.64011140	0.33057636	2.24185366
C	4.64011140	0.33057636	3.46565036
H	2.78268768	1.67935981	3.71350632
H	2.78268768	1.67935981	1.99399771
H	-1.24255629	0.20056335	3.96545466
H	-1.24255629	0.20056335	1.74204936
H	-0.80129630	-0.43500583	6.29976594
H	-0.80129630	-0.43500583	-0.59226192
C	4.12076303	0.65136269	7.21663498
C	4.12076303	0.65136269	-1.50913096
C	5.44261377	0.55307234	7.64236175
C	5.44261377	0.55307234	-1.93485772
C	6.45899301	0.37344188	6.70244874
C	6.45899301	0.37344188	-0.99494472
C	6.13822254	0.30201692	5.35232542
C	6.13822254	0.30201692	0.35517861
H	6.92270709	0.16284534	4.61451526
H	6.92270709	0.16284534	1.09298877
H	7.49599406	0.29270555	7.01679128
H	7.49599406	0.29270555	-1.30928725
H	5.67745694	0.62274080	8.70125312
H	5.67745694	0.62274080	-2.99374909
H	3.33624721	0.80954185	7.95152576
H	3.33624721	0.80954185	-2.24402174

A
42 atoms
Zero imaginary vibrational frequencies

C	0.00000000	0.00000000	0.00000000
C	0.00876799	1.36920598	0.64749002
C	-1.14190565	1.92205113	1.09238108
C	-2.36419062	1.13798031	1.06548872
C	-2.35855036	-0.20000789	0.79924355
C	-1.14221797	-0.89175496	0.43240769
C	-0.95370149	-2.24495432	0.37492256

C	-1.93931449	-3.27226696	0.63445979
C	-1.58707371	-4.58475285	0.75483721
C	-0.19710580	-5.00668887	0.76262707
C	0.76633980	-4.11429967	0.44047676
C	0.39877523	-2.74610636	-0.08321592
C	1.60611011	-1.86441580	0.25749574
C	1.42176867	-0.52465017	0.23402200
C	2.24893610	0.67593548	0.45599963
C	1.40118180	1.79164366	0.72030206
C	1.93977117	3.05324862	0.98628929
C	3.32388558	3.22493950	0.95536832
C	4.16066098	2.14800312	0.64032191
C	3.63152199	0.88029183	0.38545055
H	4.29731761	0.07913239	0.08939042
H	5.23541425	2.29925295	0.58158461
H	3.75268519	4.20429476	1.15116877
H	1.28536393	3.89724276	1.18966732
C	2.70782051	-2.78911386	0.58927347
C	2.20764507	-4.12413530	0.64381137
C	3.05083247	-5.19832696	0.94216272
C	4.39776569	-4.95675740	1.20562104
C	4.89158963	-3.64668020	1.19170655
C	4.05791787	-2.56788764	0.89005172
H	4.46643364	-1.56708431	0.91688673
H	5.93871374	-3.46291932	1.41827001
H	5.06456931	-5.78512134	1.43063959
H	2.65714764	-6.21148210	0.96804472
H	0.35947696	-2.82813626	-1.19120471
H	0.04796349	-6.00071933	1.13043032
H	-2.35371037	-5.32759079	0.96099086
H	-2.97904619	-2.98690194	0.77084605
H	-3.27780835	-0.76865741	0.91265455
H	-3.29234772	1.61936178	1.36365182
H	-1.16059917	2.90986504	1.54761230
H	-0.11487942	0.16981106	-1.09295987

7
40 atoms
Zero imaginary vibrational frequencies

C	0.00000000	0.00000000	0.00000000
C	-0.15068684	-1.44383926	-0.06536968
C	-1.36365373	-2.16932950	-0.11658044
C	-1.35660152	-3.55868804	-0.17190551
C	-0.15324866	-4.29612815	-0.17299348
C	1.04694753	-3.60607994	-0.11772204
C	1.03822845	-2.19348904	-0.06995226
C	2.39288114	-1.67390492	-0.02976816
C	2.53660870	-0.30055411	-0.03927405
C	1.31838699	0.48721038	0.00299430
C	1.61914714	1.86713547	0.05455370

C	0.58851304	2.79081211	0.11555811
C	-0.74133804	2.31887183	0.11577214
C	-1.03642749	0.96122959	0.05656336
H	-2.07524056	0.64326061	0.05584259
H	-1.55628220	3.03676359	0.16135353
H	0.78508609	3.85927273	0.15969059
C	3.08301699	1.98898023	0.00585723
C	3.64503802	0.67950592	-0.07703880
C	5.02652831	0.54379922	-0.21968034
C	5.83681467	1.68489527	-0.23551308
C	5.28006541	2.96094754	-0.11699525
C	3.89481369	3.11862479	-0.00403143
H	3.45942963	4.11274852	0.06299642
H	5.92452409	3.83611545	-0.12670084
H	6.91209175	1.57316348	-0.34682761
H	5.48225423	-0.42892857	-0.35309536
C	3.27449234	-2.86173573	0.00947481
C	2.45442599	-4.02751490	-0.06952237
C	3.01710027	-5.29991598	-0.05565802
C	4.40533483	-5.42977479	0.05701036
C	5.21288406	-4.29530436	0.17123847
C	4.65425058	-3.01216487	0.15143864
H	5.29991505	-2.15317820	0.28190426
H	6.28824835	-4.40644710	0.28208021
H	4.85624073	-6.41868117	0.06909089
H	2.38698882	-6.18371860	-0.12022161
H	-0.18315321	-5.38220033	-0.21375362
H	-2.30292537	-4.09188869	-0.21294863
H	-2.31373444	-1.64249777	-0.11445455

7 (true C_2)
40 atoms
Zero imaginary vibrational frequencies

C	0.00000000	0.00000000	0.00000000
C	-1.45183997	0.05703542	0.00000000
C	-2.29875919	0.10795966	-1.13176176
C	0.84691922	-0.05092423	-1.13176176
C	-3.67978770	0.15902223	-0.98149112
C	2.22794773	-0.10198681	-0.98149112
C	-4.28875894	0.15501809	0.29203408
C	2.83691898	-0.09798267	0.29203408
C	-3.47830854	0.09977959	1.41452798
C	2.02646858	-0.04274416	1.41452798
C	-2.07383502	0.05717084	1.26006447
C	0.62199505	-0.00013541	1.26006447
C	-1.41651633	0.02120137	2.55389506
C	-0.03532364	0.03583405	2.55389506
H	0.41940443	-0.05356273	-2.13067909
H	-1.87124440	0.11059816	-2.13067909
H	2.85635229	-0.14432609	-1.86741229

H	-4.30819226	0.20136152	-1.86741229
H	3.92023254	-0.13594723	0.37495933
H	-5.37207250	0.19298265	0.37495933
C	2.29931109	0.00353082	2.85715456
C	-3.75115105	0.05350461	2.85715456
C	1.05524507	0.07449696	3.55372876
C	-2.50708504	-0.01746153	3.55372876
C	1.06473525	0.20408784	4.94327546
C	-2.51657521	-0.14705241	4.94327546
C	2.28409247	0.22028240	5.62979138
C	-3.73593243	-0.16324698	5.62979138
C	3.49546219	0.11708642	4.94174190
C	-4.94730216	-0.06005100	4.94174190
C	3.50776165	0.01659251	3.54648471
C	-4.95960162	0.04044292	3.54648471
H	4.45178025	-0.04053891	3.00985889
H	-5.90362021	0.09757433	3.00985889
H	4.43326239	0.12789732	5.49103603
H	-5.88510236	-0.07086190	5.49103603
H	2.28435935	0.32025490	6.71208777
H	-3.73619932	-0.26321947	6.71208777
H	0.14554769	0.32756504	5.50151879
H	-1.59738766	-0.27052962	5.50151879

TS1
42 atoms
One imaginary vibrational frequency

C	0.00000000	0.00000000	0.00000000
C	0.38657900	-1.34258200	0.12871600
C	1.69597100	-1.77098700	-0.11476700
C	2.69045200	-0.82710300	-0.44795100
C	2.27908900	0.49415600	-0.49384800
C	0.96806600	0.95188200	-0.34355900
C	0.78475700	2.35339800	-0.82171700
C	-0.34272200	2.79864600	-1.52306900
C	-0.26013800	3.94495600	-2.32420200
C	0.95749800	4.59814200	-2.51855800
C	2.09879900	4.18001700	-1.80618100
C	3.46537000	4.69597900	-2.09207000
C	4.61342200	3.83819700	-2.17205700
C	4.62309700	2.41520700	-1.95651000
C	4.74141100	1.21670200	-1.73679000
C	4.99824600	-0.17302100	-1.44460200
C	4.10384400	-1.12444800	-0.82789200
C	4.60684600	-2.41445100	-0.59083800
C	5.89717400	-2.80361200	-0.93698500
C	6.75264300	-1.88828000	-1.54914200
C	6.30134600	-0.59726600	-1.79059600
H	6.96060400	0.12726900	-2.25933500
H	7.76308100	-2.17241700	-1.83018100

H 6.22935700 -3.81638500 -0.72500800
H 3.96423400 -3.13941400 -0.10188200
C 5.86278300 4.41507700 -2.49326600
C 6.00444600 5.77695100 -2.73115700
C 4.88676600 6.60961400 -2.65456000
C 3.64372100 6.06525700 -2.34210100
H 2.77818000 6.71784400 -2.26612300
H 4.98241100 7.67782000 -2.83046300
H 6.98195300 6.18470900 -2.97426300
H 6.72689300 3.76039200 -2.55584400
C 1.94050800 3.15623500 -0.87760200
H 2.78917100 2.91142700 -0.25339200
H 1.03415400 5.39351200 -3.25516000
H -1.13725900 4.27429400 -2.87572100
H -1.25743400 2.21102700 -1.51769100
H 3.01911500 1.20768800 -0.77323200
H 1.90959000 -2.83496900 -0.07584800
H -0.36483700 -2.08359200 0.39060400
H -1.04063900 0.27882000 0.14717600

TS2
42 atoms
One imaginary vibrational frequency

C 0.00000000 0.00000000 0.00000000
C 0.42685600 -1.36638300 0.03477200
C 1.77219700 -1.63131700 0.45841400
C 2.40034200 -2.87669800 0.09937700
C 1.65038900 -3.89492400 -0.44371600
C 0.26316400 -3.69987700 -0.67719500
C -0.31363800 -2.45958400 -0.50336400
H -1.34704600 -2.31283600 -0.80285100
H -0.32681300 -4.51392800 -1.09017000
H 2.12893400 -4.81305700 -0.77629900
C 3.86279900 -2.75429100 0.03767700
C 4.28352600 -1.42593600 -0.25885300
C 3.25261100 -0.40712700 -0.29231900
C 2.87168600 0.80471800 -0.32613200
C 3.13871300 2.22913100 -0.36655200
C 2.03342900 3.09483400 -0.12550500
C 0.76093300 2.36588600 -0.03936600
C 0.95049200 1.00342700 0.38883400
H 1.48572400 0.84575800 1.32580300
C -0.42979000 2.74847600 -0.61395700
C -1.45629500 1.78742900 -0.81426400
C -1.22468200 0.44962100 -0.57491700
H -1.98590400 -0.27484300 -0.84875700
H -2.40030300 2.10020400 -1.25269200
H -0.55559000 3.75879800 -0.99615300
C 2.22510400 4.47816700 -0.09801300
C 3.49682400 5.01182700 -0.31357800

C	4.58352600	4.16666200	-0.56253200
C	4.40932700	2.78310700	-0.58840300
H	5.25435200	2.12995800	-0.78563300
H	5.57009600	4.58785300	-0.73761100
H	3.64266100	6.08858000	-0.28612200
H	1.38319700	5.13572500	0.10339200
C	5.64857800	-1.16760600	-0.45966100
C	6.57886500	-2.20185100	-0.35869100
C	6.16103800	-3.50240800	-0.05637500
C	4.80679500	-3.77875000	0.13829700
H	4.48328800	-4.78783600	0.38100400
H	6.89341700	-4.30101100	0.02889800
H	7.63379400	-1.99383300	-0.51742800
H	5.97648400	-0.15996300	-0.69766000
H	2.10873200	-1.14961700	1.37713400

TS2 (true *C*$_s$)
42 atoms
One imaginary vibrational frequency

C	0.00000000	0.00000000	0.00000000
C	0.00000000	0.00000000	-1.43240000
C	1.20917900	-0.39205000	-2.09872800
C	1.20917900	-0.39205000	0.66632800
C	1.43194400	0.00738500	-3.46489900
C	1.43194400	0.00738500	2.03249900
C	0.40439300	0.55757300	-4.19720100
C	0.40439300	0.55757300	2.76480100
C	-0.86381300	0.75961600	-3.58992100
C	-0.86381300	0.75961600	2.15752100
C	-1.03980200	0.54810600	-2.23914100
C	-1.03980200	0.54810600	0.80674100
H	-1.98419000	0.82498400	-1.78018700
H	-1.98419000	0.82498400	0.34778700
H	-1.67508000	1.17885000	-4.17929900
H	-1.67508000	1.17885000	2.74689900
H	0.58102800	0.92052500	-5.20692800
H	0.58102800	0.92052500	3.77452800
C	2.86388200	0.09931500	-3.78234700
C	2.86388200	0.09931500	2.34994700
C	3.65605300	0.37685400	-2.63146400
C	3.65605300	0.37685400	1.19906400
C	2.97669600	0.36464900	-1.35126800
C	2.97669600	0.36464900	-0.08113200
H	1.68704300	-1.31345900	0.33178900
H	1.68704300	-1.31345900	-1.76418900
C	3.46372700	0.04360300	3.61042800
C	3.46372700	0.04360300	-5.04282800
C	4.83591000	0.26459500	3.74176300
C	4.83591000	0.26459500	-5.17416300
C	5.61617900	0.54820400	2.61573300

C 5.61617900 0.54820400 -4.04813300
C 5.03268400 0.60415900 1.35022500
C 5.03268400 0.60415900 -2.78262500
H 5.63979100 0.82789300 0.47803700
H 5.63979100 0.82789300 -1.91043700
H 6.68257200 0.72757400 2.72452100
H 6.68257200 0.72757400 -4.15692100
H 5.29941800 0.21396100 4.72361500
H 5.29941800 0.21396100 -6.15601500
H 2.86037100 -0.18426900 4.48553700
H 2.86037100 -0.18426900 -5.91793700

TS3
42 atoms
One imaginary vibrational frequency

C 0.00000000 0.00000000 0.00000000
C -1.29916100 -0.48696500 -0.46735700
C -1.37766500 -1.88548200 -0.46376000
C -0.14114700 -2.51250700 0.00577200
C 1.21073000 -2.00910300 -0.40610900
C 1.28682700 -0.65195200 -0.41100400
C 2.30327200 0.41174200 -0.42994100
C 1.67949100 1.65029700 -0.09212300
C 0.23383700 1.44002400 0.02742300
C -0.81806200 2.30875500 -0.07802100
C -2.13218500 1.80009200 -0.33108100
C -2.35867700 0.46236500 -0.56351400
H -3.35932100 0.12305900 -0.81834100
H -2.95820500 2.50265700 -0.40478100
H -0.66044600 3.38508800 -0.07117400
C 2.42845300 2.82134800 0.00995500
C 3.80335800 2.77604800 -0.23594000
C 4.41795300 1.57068900 -0.58915100
C 3.67557800 0.38971700 -0.68878400
H 4.17438400 -0.52719300 -0.97959600
H 5.48577600 1.54840800 -0.79083700
H 4.39684200 3.68329600 -0.15838100
H 1.94938900 3.75983800 0.27849700
C 2.10395400 -3.17786100 -0.41242000
C 1.34672300 -4.33918600 -0.07412700
C -0.06760600 -3.96969700 0.03948300
C -1.20971600 -4.71627900 -0.06238800
C -2.45907900 -4.06554000 -0.31770200
C -2.53632100 -2.71191800 -0.55579400
H -3.49323700 -2.26512100 -0.81306400
H -3.35792000 -4.67263300 -0.38829700
H -1.17342700 -5.80347500 -0.05030500
C 1.96418000 -5.58383800 0.03847200
C 3.33800800 -5.68830700 -0.19611100
C 4.08299800 -4.55778400 -0.54549900

C	3.47319200	-3.30408700	-0.65558900
H	4.07091800	-2.44575200	-0.93798800
H	5.14894300	-4.65105600	-0.73586000
H	3.82828600	-6.65449300	-0.11043400
H	1.38568400	-6.46416200	0.30778100
H	-0.11380800	-1.83218800	1.22392300
H	-0.05131000	-0.67184900	1.22080200

TS3 (true C_s)
42 atoms
One imaginary vibrational frequency

C	0.00000000	0.00000000	0.00000000
C	0.00000000	0.00000000	-2.51631200
C	-1.26087100	0.49264500	-0.55774100
C	-1.26087100	0.49264500	-1.95857100
C	1.32914600	0.38592000	-1.93767500
C	1.32914600	0.38592000	-0.57863700
C	2.28576900	0.38229900	0.53933600
C	2.28576900	0.38229900	-3.05564800
C	1.58767300	0.05617200	1.74045800
C	1.58767300	0.05617200	-4.25677000
C	0.15397000	-0.03361200	1.45069600
C	0.15397000	-0.03361200	-3.96700800
C	-0.94197400	0.09192700	2.26037200
C	-0.94197400	0.09192700	-4.77668400
C	-2.22065400	0.37084900	1.68034600
C	-2.22065400	0.37084900	-4.19665800
C	-2.36897100	0.60825000	0.33233900
C	-2.36897100	0.60825000	-2.84865100
H	-3.34460500	0.88315900	-0.06004100
H	-3.34460500	0.88315900	-2.45627100
H	-3.08278400	0.46016900	2.33619300
H	-3.08278400	0.46016900	-4.85250500
H	-0.84386300	0.08076100	3.34365000
H	-0.84386300	0.08076100	-5.85996200
C	2.26808500	-0.06265500	2.95092400
C	2.26808500	-0.06265500	-5.46723600
C	3.64791500	0.15607800	2.98279400
C	3.64791500	0.15607800	-5.49910600
C	4.33577400	0.49678700	1.81387700
C	4.33577400	0.49678700	-4.33018900
C	3.66255900	0.61135200	0.59342700
C	3.66255900	0.61135200	-3.10973900
H	4.21715200	0.88806200	-0.29532200
H	4.21715200	0.88806200	-2.22099000
H	5.40700900	0.67665000	1.85147400
H	5.40700900	0.67665000	-4.36778600
H	4.18754600	0.06618900	3.92190300
H	4.18754600	0.06618900	-6.43821500
H	1.73285700	-0.32281400	3.86099300

H 1.73285700 -0.32281400 -6.37730500
H -0.03714900 -1.21965900 -1.83961700
H -0.03714900 -1.21965900 -0.67669500

H_2
2 atoms
Zero imaginary vibrational frequencies

H 0.00000000 0.00000000 0.00000000
H -0.74311277 0.00000000 0.00000000

H_2 (true $D_{\infty h}$)
2 atoms
Zero imaginary vibrational frequencies

H 0.00000000 0.00000000 0.00000000
H 0.00000000 0.00000000 -0.74311314

B3LYP/6-31G(d) Thermochemistry Data (hartrees)

	E	*H*	*G*	*qG*
**6**-C_2	-999.714797	-999.329513	-999.477476	-999.475041
**6**-C_2 (C_2)	-999.714748	-999.329599	-999.476678	-999.474193
**6**-C_s	-999.706300	-999.321024	-999.469848	-999.466607
**6**-C_s (C_s)	-999.706318	-999.321026	-999.469651	-999.466576
**A**	-999.721811	-999.337649	-999.480800	-999.478400
**7**	-998.627967	-998.266427	-998.405960	-998.404037
**7** (C_2)	-998.627911	-998.266401	-998.404984	-998.403029
**TS1**	-999.705241	-999.321201	-999.465306	-999.463356
**TS2**	-999.650583	-999.269228	-999.414024	-999.411958
**TS2** (C_s)	-999.650579	-999.269265	-999.414128	-999.412041
**TS3**	-999.676747	-999.300995	-999.443057	-999.440354
**TS3** (C_s)	-999.676802	-999.301000	-999.442727	-999.440296
H_2	-1.168716	-1.153114	-1.180332	-1.180332
H_2 ($D_{\infty h}$)	-1.168698	-1.153095	-1.180314	-1.180314

3.2. ωB97X-D/6-311+G(d,p) Coordinates and Thermochemistry Data

6-C_2
42 atoms
Zero imaginary vibrational frequencies

C	0.00000000	0.00000000	0.00000000
C	-0.47925600	-1.25669600	0.37587000
C	0.29850300	-2.05528400	1.20300400
C	1.50308300	-1.54428700	1.69052100
C	2.05200400	-0.36266900	1.22717600
C	1.26805200	0.42721300	0.37761300
H	1.66194100	1.35530500	-0.02250300
C	3.46064700	-0.04587200	1.57428600
C	4.46505700	-1.04623000	1.64519400
C	4.26107100	-2.44934700	1.38717300
C	4.18535200	-3.64429600	1.21766600
C	4.20723500	-5.06194700	0.95969100
C	3.08123400	-5.92344200	1.02365400
C	1.72617800	-5.43117500	1.37772900
C	0.84934100	-6.12353600	2.22143600
C	-0.35115300	-5.54037300	2.61061100
C	-0.66613600	-4.22724300	2.25369700
C	0.20513700	-3.52612200	1.43155500
C	1.33159000	-4.18299900	0.93242900
H	1.96998900	-3.64011800	0.25040200
H	-1.54540200	-3.74488000	2.66620100
H	-1.01947600	-6.08914600	3.26475600
H	1.12068100	-7.10015600	2.60778200
C	3.26952900	-7.28152000	0.76074300
C	4.51551900	-7.80332800	0.44878900
C	5.61829200	-6.96080000	0.38993100
C	5.45923500	-5.60825300	0.64029000
H	6.31412500	-4.94384000	0.59797700
H	6.59886800	-7.35322700	0.14729500
H	4.62289400	-8.86265400	0.24587900
H	2.40670500	-7.93776300	0.78651300
C	5.77538200	-0.66739800	1.97297100
C	6.10749800	0.65321400	2.22366900
C	5.12479900	1.63237700	2.15306400
C	3.82324100	1.27693500	1.83349100
H	3.05413000	2.04059400	1.80058000
H	5.36804700	2.66918500	2.35498100
H	7.12870400	0.91486800	2.47490500
H	6.53541000	-1.43802700	2.02388900
H	2.07193000	-2.15677400	2.37532700
H	-1.41492900	-1.62819300	-0.02720000
H	-0.59548400	0.62195000	-0.65897600

6-C_2 (true C_2)
42 atoms
Zero imaginary vibrational frequencies

C	0.00000000	0.00000000	0.00000000
C	-5.43106416	2.86130362	0.00000000
C	-1.26921643	0.42316852	-0.40169098
C	-4.16184773	2.43813510	-0.40169098
C	-1.98411034	1.28349017	0.42048160
C	-3.44695382	1.57781345	0.42048160
C	-1.37867517	1.75274603	1.58821119
C	-4.05238898	1.10855759	1.58821119
C	-0.18343627	1.24552210	2.06413589
C	-5.24762789	1.61578152	2.06413589
C	0.52106577	0.36357743	1.23623606
C	-5.95212993	2.49772620	1.23623606
H	1.45605132	-0.07121939	1.57303924
H	-6.88711547	2.93252301	1.57303924
C	0.23744295	1.57881355	3.44836062
C	-5.66850710	1.28249007	3.44836062
C	-0.69115794	1.68684240	4.51574148
C	-4.73990621	1.17446122	4.51574148
C	-2.11385072	1.48989635	4.40170630
C	-3.31721344	1.37140727	4.40170630
H	-3.50388246	0.39875132	2.19075505
H	-1.92718170	2.46255230	2.19075505
H	-3.71822904	2.82953780	-1.31049404
H	-1.71283511	0.03176583	-1.31049404
H	-5.98821710	3.54580491	-0.62987744
H	0.55715295	-0.68450128	-0.62987744
C	-7.02217636	1.08209839	3.72440049
C	1.59111221	1.77920524	3.72440049
C	-7.47544389	0.78735667	5.00118172
C	2.04437973	2.07394696	5.00118172
C	-6.56644975	0.68242183	6.04617672
C	1.13538559	2.17888180	6.04617672
C	-5.21721497	0.87283011	5.80001506
C	-0.21384919	1.98847351	5.80001506
H	-4.50073363	0.79485953	6.60912566
H	-0.93033052	2.06644410	6.60912566
H	-6.90483962	0.45133428	7.04943533
H	1.47377546	2.40996935	7.04943533
H	-8.53382360	0.63271140	5.17671705
H	3.10275944	2.22859223	5.17671705
H	-7.73200776	1.14329242	2.90695602
H	2.30094361	1.71801121	2.90695602

6-C_s
42 atoms
Zero imaginary vibrational frequencies

C 0.00000000 0.00000000 0.00000000
C -0.29916541 -1.29869945 -0.40617916
C 0.70313059 -2.11833745 -0.92545582
C 1.95875758 -1.53997910 -1.11686520
C 2.31693391 -0.32236668 -0.56784680
C 1.30731667 0.47050711 -0.01316765
H 1.54225824 1.42324465 0.44798806
C 3.76158132 0.00513686 -0.46270041
C 4.74651679 -0.98976693 -0.21888015
C 4.51257400 -2.41079261 -0.12952119
C 4.46288329 -3.62075603 -0.12888097
C 4.58366370 -5.05574892 -0.21464220
C 3.52233102 -5.97040151 -0.45057536
C 2.10846847 -5.52736678 -0.54698610
C 1.04750777 -6.22380073 0.04034001
C -0.21569793 -5.64606685 0.06886801
C -0.41207546 -4.33430346 -0.35698846
C 0.64365538 -3.61120891 -0.91266864
C 1.84342474 -4.29496758 -1.11629276
H 2.65911938 -3.81554698 -1.62670121
H -1.37392310 -3.86524337 -0.18481187
H -1.04205753 -6.18954168 0.51299423
H 1.21323235 -7.18456983 0.51480740
C 3.82763299 -7.33017555 -0.54173184
C 5.12206534 -7.80492814 -0.40117537
C 6.15863826 -6.91222144 -0.15943538
C 5.88667625 -5.55801446 -0.07108502
H 6.68958134 -4.85459845 0.11491796
H 7.17594364 -7.26731362 -0.04378088
H 5.32092507 -8.86714326 -0.48534654
H 3.02354647 -8.02868804 -0.74494419
C 6.08497787 -0.59254695 -0.07327240
C 6.46391354 0.73538629 -0.16690692
C 5.50214382 1.70651800 -0.41567001
C 4.17439326 1.33566111 -0.55864236
H 3.42936857 2.09495481 -0.76803471
H 5.78472266 2.74917084 -0.50391037
H 7.50603596 1.00875135 -0.05020357
H 6.82900898 -1.35677323 0.11746970
H 2.74044936 -2.09673108 -1.60002071
H -1.30121519 -1.68076127 -0.24903663
H -0.78606974 0.62014560 0.41598029

6-C_s (true C_s)
42 atoms
Zero imaginary vibrational frequencies

C 0.00000000 0.00000000 0.00000000
C 0.00000000 0.00000000 5.64984079

C -0.25855216 -0.40922942 1.30624496
C -0.25855216 -0.40922942 4.34359583
C 0.75708237 -0.97439128 2.07780928
C 0.75708237 -0.97439128 3.57203150
C 1.97992491 -1.20474592 1.44568895
C 1.97992491 -1.20474592 4.20415184
C 2.30607624 -0.65448401 0.21929608
C 2.30607624 -0.65448401 5.43054471
C 1.28517399 -0.05569530 -0.52535442
C 1.28517399 -0.05569530 6.17519521
H 1.49743347 0.40679460 -1.48278054
H 1.49743347 0.40679460 7.13262133
C 3.73887917 -0.59319524 -0.16613893
C 3.73887917 -0.59319524 5.81597972
C 4.76753425 -0.38178847 0.79079576
C 4.76753425 -0.38178847 4.85904502
C 4.59085889 -0.28829780 2.21947253
C 4.59085889 -0.28829780 3.43036826
H 2.76614182 -1.72296727 3.68585234
H 2.76614182 -1.72296727 1.96398845
H -1.23648834 -0.21822630 3.91705731
H -1.23648834 -0.21822630 1.73278348
H -0.79552607 0.45201377 6.23151245
H -0.79552607 0.45201377 -0.58167166
C 4.09768611 -0.69740535 7.16165334
C 4.09768611 -0.69740535 -1.51181255
C 5.41435420 -0.59429933 7.58214684
C 5.41435420 -0.59429933 -1.93230605
C 6.41934523 -0.37790571 6.64768370
C 6.41934523 -0.37790571 -0.99784291
C 6.09375735 -0.27602897 5.30633472
C 6.09375735 -0.27602897 0.34350607
H 6.87135586 -0.10966104 4.57033842
H 6.87135586 -0.10966104 1.07950237
H 7.45353720 -0.29276675 6.95995821
H 7.45353720 -0.29276675 -1.31011742
H 5.65485745 -0.68821225 8.63486275
H 5.65485745 -0.68821225 -2.98502196
H 3.31824795 -0.88140987 7.89258913
H 3.31824795 -0.88140987 -2.24274834

A
42 atoms
Zero imaginary vibrational frequencies

C 0.00000000 0.00000000 0.00000000
C 0.01765466 1.32551216 0.71829778
C -1.10929885 1.85477162 1.21178554
C -2.34752086 1.09050666 1.13007932
C -2.35610058 -0.21529763 0.78837560
C -1.13143219 -0.90113061 0.40741122

C	-0.94553479	-2.23711451	0.34564454
C	-1.94300202	-3.26373796	0.60743610
C	-1.58967062	-4.55368105	0.78902854
C	-0.19158602	-4.96121548	0.85021747
C	0.75779293	-4.08881045	0.48758852
C	0.39871243	-2.74947442	-0.09289091
C	1.59414592	-1.86192210	0.22798168
C	1.41143711	-0.53520276	0.20400671
C	2.24620834	0.65413221	0.45343857
C	1.41433134	1.74478144	0.78755001
C	1.95197846	2.98997803	1.08882510
C	3.32836858	3.16429594	1.02037199
C	4.15097447	2.11064009	0.62710000
C	3.61765524	0.86002357	0.33539775
H	4.26882448	0.07732134	-0.02673554
H	5.21902937	2.27052785	0.53231641
H	3.76283887	4.13220842	1.24273807
H	1.30384526	3.82002112	1.34749523
C	2.69821565	-2.77362089	0.58714872
C	2.20129292	-4.09138901	0.69508787
C	3.03251704	-5.15036719	1.04145602
C	4.37293835	-4.90043291	1.29795838
C	4.86717698	-3.59927104	1.22702966
C	4.04067087	-2.53803554	0.87759573
H	4.45145052	-1.54078676	0.85694959
H	5.91132319	-3.40893119	1.44819682
H	5.03603721	-5.71605877	1.56287103
H	2.63522900	-6.15705120	1.11131603
H	0.36793911	-2.87512459	-1.19238805
H	0.05802341	-5.92655415	1.27754002
H	-2.35466007	-5.29506468	0.99167904
H	-2.98639777	-2.98039984	0.68633699
H	-3.28269083	-0.77548887	0.84299532
H	-3.27119236	1.56925965	1.43600864
H	-1.10965587	2.80687969	1.73162442
H	-0.12400870	0.22626123	-1.07710919

7
40 atoms
Zero imaginary vibrational frequencies

C	0.00000000	0.00000000	0.00000000
C	-0.15050500	-1.44144400	-0.06328100
C	-1.35874900	-2.16342300	-0.11552900
C	-1.35064500	-3.54529600	-0.17016700
C	-0.15034900	-4.27883700	-0.16983800
C	1.03947000	-3.58834000	-0.11414900
C	1.02979100	-2.18420200	-0.06659600
C	2.38144500	-1.66400300	-0.02805500
C	2.52361300	-0.30603400	-0.04112400
C	1.30853500	0.48207000	0.00170100

C	1.60864200	1.85325200	0.05622000
C	0.58824500	2.77520500	0.11891800
C	-0.73791900	2.30666200	0.11915900
C	-1.03241200	0.95664000	0.05884300
H	-2.06905900	0.63975900	0.05944900
H	-1.55032600	3.02298600	0.16686900
H	0.78807600	3.84009300	0.16498100
C	3.07261500	1.97322400	0.00954900
C	3.63094000	0.67520400	-0.07421600
C	5.00474800	0.53864100	-0.21804700
C	5.81007500	1.67574900	-0.23181700
C	5.25472800	2.94506400	-0.11240100
C	3.87570900	3.10075900	0.00002800
H	3.43807100	4.09065500	0.06892500
H	5.89757500	3.81784200	-0.12171800
H	6.88271600	1.56691400	-0.34360300
H	5.46235800	-0.43060000	-0.34759900
C	3.26171000	-2.85266100	0.00706400
C	2.44749400	-4.00815400	-0.06743300
C	3.00192900	-5.27664700	-0.04826300
C	4.38382200	-5.40677500	0.06306600
C	5.18889200	-4.27813800	0.17104100
C	4.63441500	-3.00004700	0.14852000
H	5.28148200	-2.14438300	0.26972000
H	6.26122700	-4.39127000	0.28147100
H	4.83345800	-6.39298100	0.08052100
H	2.37028800	-6.15624400	-0.10740700
H	-0.17624600	-5.36221300	-0.21033800
H	-2.29397300	-4.07795900	-0.21239400
H	-2.30694200	-1.63809400	-0.11496600

7 (true C_2)
40 atoms
Zero imaginary vibrational frequencies

C	0.00000000	0.00000000	0.00000000
C	-1.44949430	0.05741277	0.00000000
C	-2.29280619	0.10909939	-1.12704613
C	0.84331188	-0.05168662	-1.12704613
C	-3.66631565	0.16150480	-0.97613155
C	2.21682135	-0.10409203	-0.97613155
C	-4.27092183	0.15925165	0.29410772
C	2.82142752	-0.10183887	0.29410772
C	-3.46069014	0.10346160	1.40591603
C	2.01119584	-0.04604883	1.40591603
C	-2.06492551	0.05791231	1.25110299
C	0.61543121	-0.00049954	1.25110299
C	-1.40750675	0.02039683	2.54208217
C	-0.04198755	0.03701594	2.54208217
H	0.41866404	-0.05315775	-2.12445754
H	-1.86815834	0.11057052	-2.12445754

H 2.84442108 -0.14569025 -1.85918276
H -4.29391538 0.20310302 -1.85918276
H 3.90158853 -0.14127689 0.38132354
H -5.35108283 0.19868966 0.38132354
C 2.28280254 -0.00033212 2.84896661
C -3.73229684 0.05774489 2.84896661
C 1.04971728 0.07260042 3.54030773
C -2.49921158 -0.01518765 3.54030773
C 1.05652879 0.20793262 4.92182563
C -2.50602309 -0.15051985 4.92182563
C 2.27124956 0.22692838 5.60395436
C -3.72074386 -0.16951561 5.60395436
C 3.47644613 0.12149081 4.91852802
C -4.92594043 -0.06407804 4.91852802
C 3.48803639 0.01601455 3.53007395
C -4.93753069 0.04139823 3.53007395
H 4.42754332 -0.04281521 2.99152189
H -5.87703762 0.10022798 2.99152189
H 4.41126168 0.13584107 5.46713008
H -5.86075599 -0.07842830 5.46713008
H 2.27407021 0.33197019 6.68285261
H -3.72356451 -0.27455742 6.68285261
H 0.13996235 0.32669433 5.47983559
H -1.58945665 -0.26928156 5.47983559

TS1
42 atoms
One imaginary vibrational frequency

C 0.00000000 0.00000000 0.00000000
C 0.39122900 -1.33335200 0.14487900
C 1.68854700 -1.76147100 -0.12812800
C 2.66492300 -0.82924000 -0.50931400
C 2.25177500 0.48218900 -0.57985500
C 0.95207200 0.93533900 -0.39260300
C 0.76214900 2.32399400 -0.89873200
C -0.33632300 2.71455300 -1.65784600
C -0.24052700 3.82278900 -2.49976400
C 0.97049400 4.48020200 -2.67083200
C 2.07795800 4.11423500 -1.89553800
C 3.44201800 4.64613300 -2.15394000
C 4.58230800 3.80329300 -2.23785000
C 4.58526300 2.37295900 -2.05229300
C 4.69733500 1.18419900 -1.84809600
C 4.94784300 -0.21040300 -1.55724800
C 4.06851800 -1.13926800 -0.91783700
C 4.56280400 -2.42498500 -0.67903200
C 5.83543400 -2.82547600 -1.05175900
C 6.67759500 -1.92753200 -1.69156300
C 6.23118500 -0.64061400 -1.93161400
H 6.88235300 0.07394500 -2.42105600

H 7.67543100 -2.22119500 -1.99526000
H 6.16508000 -3.83586400 -0.83929800
H 3.92871900 -3.14119000 -0.17198100
C 5.82898700 4.38237000 -2.52160600
C 5.96865800 5.74520900 -2.72053900
C 4.85254600 6.56838700 -2.64082300
C 3.61173200 6.01585500 -2.36306200
H 2.74291600 6.66024500 -2.28632100
H 4.94800500 7.63799300 -2.78798300
H 6.94562600 6.16161200 -2.93628400
H 6.69409000 3.73314600 -2.58624400
C 1.90489300 3.13343400 -0.93586300
H 2.73565400 2.91166700 -0.28015000
H 1.06982700 5.24349500 -3.43489000
H -1.09534600 4.11124700 -3.10105500
H -1.23554900 2.10791300 -1.66667200
H 2.97094200 1.19682300 -0.90338700
H 1.90425800 -2.82134800 -0.06609300
H -0.34726500 -2.06826100 0.44577600
H -1.03285800 0.28338400 0.17181000

TS2
42 atoms
One imaginary vibrational frequency

C 0.00000000 0.00000000 0.00000000
C 0.42488700 -1.35951000 0.03707000
C 1.75669400 -1.62350600 0.45913000
C 2.38019300 -2.86361500 0.09654700
C 1.64576100 -3.86748100 -0.46481500
C 0.26160500 -3.67239000 -0.70579500
C -0.31102100 -2.44377800 -0.52114500
H -1.34158700 -2.29480600 -0.81972600
H -0.32326300 -4.47938700 -1.13189300
H 2.13049800 -4.77525600 -0.80814400
C 3.84162100 -2.73771700 0.02953900
C 4.24593700 -1.42541300 -0.29377300
C 3.20975600 -0.41045800 -0.33041600
C 2.83615100 0.78764300 -0.36364200
C 3.10949800 2.21222500 -0.39665000
C 2.02421200 3.07472400 -0.13553400
C 0.75046000 2.34944100 -0.04946200
C 0.93825100 0.99597600 0.38694100
H 1.49904000 0.83964700 1.30647100
C -0.41655800 2.72686200 -0.64794100
C -1.44056300 1.76716800 -0.85410600
C -1.21440300 0.44263200 -0.59833000
H -1.97282600 -0.28145600 -0.87021200
H -2.37498600 2.07542500 -1.30869800
H -0.52890000 3.73001000 -1.04544300
C 2.22142500 4.44937900 -0.08911500

C	3.49031100	4.97198800	-0.30815700
C	4.56235200	4.12563200	-0.57917800
C	4.37708000	2.74983100	-0.62240800
H	5.21140200	2.09234400	-0.83516300
H	5.54775100	4.54111600	-0.75612100
H	3.64662600	6.04391300	-0.26654500
H	1.38714000	5.10730700	0.12797600
C	5.59721700	-1.15541800	-0.51143000
C	6.53325300	-2.17500300	-0.39682100
C	6.13073600	-3.46568300	-0.06312700
C	4.78667700	-3.74961800	0.14694700
H	4.47256500	-4.75328800	0.41157700
H	6.86980700	-4.25271100	0.03461900
H	7.58243400	-1.96325100	-0.56791500
H	5.91103700	-0.15194100	-0.77249200
H	2.11443900	-1.12972900	1.36072900

TS2 (true C_s)
42 atoms
One imaginary vibrational frequency

C	0.00000000	0.00000000	0.00000000
C	0.00000000	0.00000000	-1.42489400
C	1.19694000	-0.38965600	-2.08600200
C	1.19694000	-0.38965600	0.66110800
C	1.41722800	0.01398100	-3.44481300
C	1.41722800	0.01398100	2.01991900
C	0.40921400	0.58322300	-4.16762500
C	0.40921400	0.58322300	2.74273100
C	-0.85669800	0.79130500	-3.56182800
C	-0.85669800	0.79130500	2.13693400
C	-1.03326700	0.56739500	-2.22412600
C	-1.03326700	0.56739500	0.79923200
H	-1.97549900	0.84160400	-1.76544400
H	-1.97549900	0.84160400	0.34055000
H	-1.66116100	1.22363000	-4.14559100
H	-1.66116100	1.22363000	2.72069700
H	0.59600200	0.95769800	-5.16848700
H	0.59600200	0.95769800	3.74359300
C	2.84890500	0.10731900	-3.75871000
C	2.84890500	0.10731900	2.33381600
C	3.62248700	0.40881100	-2.61825100
C	3.62248700	0.40881100	1.19335700
C	2.93615500	0.40268200	-1.34006600
C	2.93615500	0.40268200	-0.08482800
H	1.69801400	-1.29381600	0.32025700
H	1.69801400	-1.29381600	-1.74515100
C	3.45078300	0.03111900	3.58398100
C	3.45078300	0.03111900	-5.00887500
C	4.81615600	0.25929600	3.70720100
C	4.81615600	0.25929600	-5.13209500

C 5.58145600 0.57044300 2.58626200
C 5.58145600 0.57044300 -4.01115600
C 4.99038500 0.64469300 1.33160900
C 4.99038500 0.64469300 -2.75650300
H 5.58595300 0.88857200 0.46018100
H 5.58595300 0.88857200 -1.88507500
H 6.64434600 0.75504500 2.69118200
H 6.64434600 0.75504500 -4.11607600
H 5.28777000 0.19307200 4.68103500
H 5.28777000 0.19307200 -6.10592900
H 2.85505300 -0.21666400 4.45562500
H 2.85505300 -0.21666400 -5.88051900

TS3
42 atoms
One imaginary vibrational frequency

C 0.00000000 0.00000000 0.00000000
C -1.28965500 -0.48282900 -0.45434300
C -1.36691400 -1.86880000 -0.44943900
C -0.13849700 -2.48834600 0.00766700
C 1.20288200 -1.99028800 -0.40316800
C 1.27776400 -0.64534600 -0.40738800
C 2.29544100 0.41596500 -0.41032300
C 1.67975600 1.64111100 -0.06258700
C 0.23168600 1.43430500 0.04826300
C -0.80741000 2.29844400 -0.04946200
C -2.12680600 1.79013900 -0.29039800
C -2.35384200 0.46702100 -0.52958000
H -3.35396500 0.12880100 -0.77615900
H -2.95161400 2.49136100 -0.34728400
H -0.64784800 3.37160600 -0.04115600
C 2.42569200 2.80336400 0.06086200
C 3.79562100 2.75502500 -0.17703800
C 4.40460600 1.55775400 -0.54164200
C 3.66143700 0.38662500 -0.66098300
H 4.15563900 -0.52735300 -0.95787600
H 5.47067900 1.53541200 -0.73671900
H 4.39182700 3.65559400 -0.08455400
H 1.94832800 3.73688100 0.33817700
C 2.09782300 -3.15711300 -0.39694900
C 1.35087700 -4.30494700 -0.04308900
C -0.06569500 -3.93912000 0.06481100
C -1.19386900 -4.68388400 -0.02667500
C -2.44958300 -4.03458700 -0.26883600
C -2.52990100 -2.69598400 -0.51618700
H -3.48729800 -2.25104500 -0.76305600
H -3.34686200 -4.64100800 -0.31855200
H -1.15380700 -5.76805300 -0.01150600
C 1.96495900 -5.54092800 0.09009400
C 3.33261400 -5.64419900 -0.14364100

C 4.07015100 -4.52305000 -0.51270500
C 3.45968400 -3.27849200 -0.64191300
H 4.05146200 -2.42519900 -0.94081900
H 5.13299200 -4.61805200 -0.70325100
H 3.82594600 -6.60410300 -0.04262900
H 1.38824700 -6.41495400 0.37232600
H -0.10088700 -1.79910400 1.21657300
H -0.04083300 -0.68097300 1.21332000

TS3 (true *C_s*)
42 atoms
One imaginary vibrational frequency

C 0.00000000 0.00000000 0.00000000
C 0.00000000 0.00000000 -2.49168600
C -1.25286700 -0.47798200 -0.55179500
C -1.25286700 -0.47798200 -1.93989100
C 1.31893900 -0.38578800 -1.91928600
C 1.31893900 -0.38578800 -0.57240000
C 2.27639500 -0.36819600 0.54378800
C 2.27639500 -0.36819600 -3.03547400
C 1.58712300 -0.02852600 1.73128600
C 1.58712300 -0.02852600 -4.22297200
C 0.15142200 0.05581900 1.44466800
C 0.15142200 0.05581900 -3.93635400
C -0.93211800 -0.05702600 2.25046200
C -0.93211800 -0.05702600 -4.74214800
C -2.21730700 -0.32049400 1.67081200
C -2.21730700 -0.32049400 -4.16249800
C -2.36683700 -0.56717200 0.33798600
C -2.36683700 -0.56717200 -2.82967200
H -3.34275600 -0.83085700 -0.05393500
H -3.34275600 -0.83085700 -2.43775100
H -3.07898600 -0.38755700 2.32525900
H -3.07898600 -0.38755700 -4.81694500
H -0.83211800 -0.04295000 3.33071100
H -0.83211800 -0.04295000 -5.82239700
C 2.26399600 0.11140000 2.93306000
C 2.26399600 0.11140000 -5.42474600
C 3.63826200 -0.10313600 2.96281100
C 3.63826200 -0.10313600 -5.45449700
C 4.31995600 -0.45961800 1.80280700
C 4.31995600 -0.45961800 -4.29449300
C 3.64634700 -0.59411300 0.59184100
C 3.64634700 -0.59411300 -3.08352700
H 4.19618800 -0.88256100 -0.29263900
H 4.19618800 -0.88256100 -2.19904700
H 5.38881200 -0.63599800 1.84114900
H 5.38881200 -0.63599800 -4.33283500
H 4.18070700 0.00217700 3.89534900

H 4.18070700 0.00217700 -6.38703500
H 1.73014700 0.38284700 3.83724900
H 1.73014700 0.38284700 -6.32893500
H -0.02430600 1.21075000 -1.80584800
H -0.02430600 1.21075000 -0.68583800

H_2
2 atoms
Zero imaginary vibrational frequencies

H 0.00000000 0.00000000 0.00000000
H -0.74440000 0.00000000 0.00000000

H_2 (true $D_{\infty h}$)
2 atoms
Zero imaginary vibrational frequencies

H 0.00000000 0.00000000 0.00000000
H 0.00000000 0.00000000 0.74440000

ωB97X-D/6-311+G(d,p) Thermochemistry Data (hartrees)

	E	*H*	*G*	*qG*
**6**-C_2	-1000.237343	-999.850497	-999.997682	-999.995137
**6**-C_2 (C_2)	-1000.237272	-999.850509	-999.996713	-999.994205
**6**-C_s	-1000.227379	-999.840440	-999.988317	-999.984986
**6**-C_s (C_s)	-1000.227413	-999.840284	-999.987415	-999.984610
**A**	-1000.248721	-999.862634	-1000.005713	-1000.002896
**7**	-999.133650	-998.770646	-998.910371	-998.908107
**7** (C_2)	-999.133505	-998.770585	-998.909648	-998.907185
**TS1**	-1000.225748	-999.840221	-999.983703	-999.981591
**TS2**	-1000.165611	-999.782689	-999.926783	-999.924611
**TS2** (C_s)	-1000.165687	-999.782692	-999.926622	-999.924539
**TS3**	-1000.199525	-999.821481	-999.962132	-999.960020
**TS3** (C_s)	-1000.199510	-999.821614	-999.962334	-999.960253
H_2	-1.176087	-1.160516	-1.187739	-1.187740
H_2 ($D_{\infty h}$)	-1.176068	-1.160497	-1.187721	-1.187721

3.3. Alternative Mechanisms

Alternative mechanism geometries were optimized using the density functional B3LYP with the unrestricted form for diradical intermediates and transition state structures, and the 6-31G(d) basis set. Vibrational frequency calculations confirmed the geometries to be minima and saddle points respectively, and Truhlar's quasiharmonic correction[1] was used to calculate free energies. Three alternative mechanisms were explored: (1) a Diels–Alder reaction directly occurring from **6**-C_2 to form **A**-*trans*, (2) an asymmetric diradical pathway beginning with **6**-C_2, and (3) an asymmetric diradical pathway beginning with **6**-C_s. The energy barriers for each of these alternative mechanisms were calculated to be too high energy to be feasible, as shown in Figure S1.

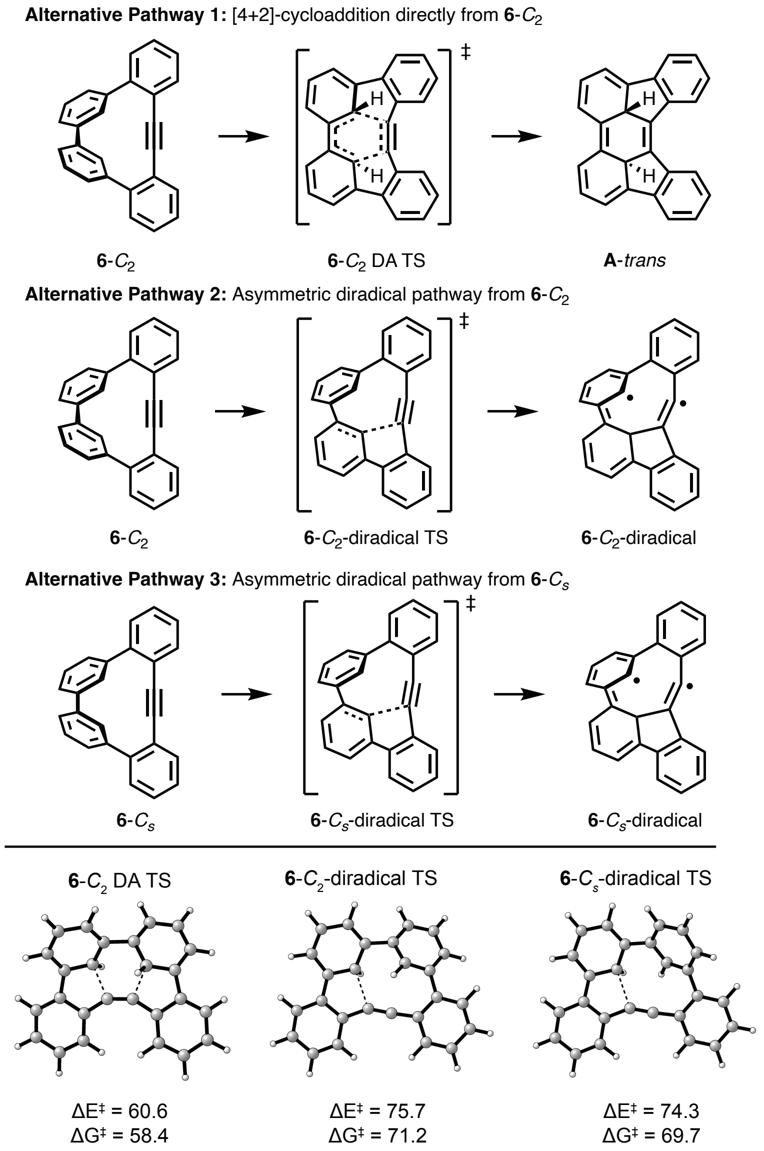

Figure S1. (top) Pathways for three alternative mechanisms that were investigated. (bottom) Computed transition state structures and their energies relative to **6**-C_2. Free energies are calculated at 493.15 K and 1 atm, and energies are presented in kcal/mol units.

[1] Ribeiro, R. F.; Marenich, A. V.; Cramer, C. J.; Truhlar, D. G. Use of Solution-Phase Vibrational Frequencies in Continuum Models for the Free Energy of Solvation *J. Phys. Chem. B* **2011**, 115, 49, 14556–14562.

3.3.1. Coordinates and Thermochemistry Data for Alternative Mechanisms

6-*C*$_2$-diradical (triplet)
42 atoms
Zero imaginary vibrational frequencies

C	0.00000000	0.00000000	0.00000000
C	-0.30626291	-1.36333635	0.08192744
C	0.62110153	-2.25881732	0.64461121
C	1.79124104	-1.71380560	1.19365085
C	2.22037066	-0.41885209	0.87391327
C	1.28510856	0.46301782	0.31492845
H	1.57877355	1.47430192	0.04392315
C	3.68883901	-0.25992931	0.77826352
C	4.40825141	-1.41219877	0.28645235
C	3.72356580	-2.64613415	0.09899876
C	3.31649374	-3.90944165	0.05998249
C	4.11580842	-5.13817184	0.19506305
C	3.27347155	-6.28462349	0.19023076
C	1.89748074	-5.87692039	0.08790938
C	0.72733027	-6.59870669	0.24240949
C	-0.48842093	-5.92027420	0.42088844
C	-0.51487536	-4.50900983	0.60714136
C	0.60079672	-3.71947721	0.47037002
C	1.84189985	-4.38435452	-0.15670691
H	1.67332901	-4.24997959	-1.24699911
H	-1.44536470	-4.04696949	0.93211636
H	-1.40701882	-6.48620069	0.54748960
H	0.75276571	-7.68413190	0.31562224
C	3.83362996	-7.56714078	0.33444280
C	5.20925667	-7.69449814	0.48540884
C	6.03905718	-6.55751403	0.49851238
C	5.49902535	-5.28182372	0.35100241
H	6.14212057	-4.40583640	0.37064134
H	7.11173379	-6.67735296	0.62646433
H	5.65040159	-8.68154837	0.59811354
H	3.19693518	-8.44843288	0.33294246
C	5.77530482	-1.25800345	-0.04118844
C	6.42423494	-0.04073869	0.14107825
C	5.72926411	1.05802256	0.65655076
C	4.37200071	0.94413879	0.96252334
H	3.82884346	1.80551013	1.34390926
H	6.24059850	2.00479980	0.80887616
H	7.47597539	0.05103655	-0.11744579
H	6.31748292	-2.10629243	-0.44945474
H	2.40613736	-2.32983819	1.83447211
H	-1.22975762	-1.74003664	-0.35095543
H	-0.72594060	0.68648500	-0.42834369

6-C_s-diradical (triplet)
42 atoms
Zero imaginary vibrational frequencies

C	0.00000000	0.00000000	0.00000000
C	0.10081021	1.48396968	0.32286332
C	-0.99389214	2.31393208	0.15221196
C	-2.26877486	1.75431811	-0.02444085
C	-2.46372895	0.35821673	0.16629408
C	-1.41938751	-0.52440522	0.30548958
C	-1.55460489	-1.91845294	0.73323057
C	-2.38783085	-2.38501209	1.76750855
C	-2.13395833	-3.63026712	2.35263858
C	-0.98422466	-4.36040876	2.02252593
C	-0.15582895	-3.90365276	0.98603876
C	-0.57017571	-2.78515453	0.26482175
H	-0.00934744	-2.50145257	-0.61629737
C	1.24495510	-4.29308356	0.72943086
C	2.17061098	-3.21110998	0.47764837
C	3.50422946	-3.55299685	0.13267769
C	3.92172653	-4.87730795	0.08481061
C	3.02822767	-5.91165222	0.38635449
C	1.70260272	-5.61182571	0.70257481
H	0.99563884	-6.41579672	0.89402463
H	3.35880880	-6.94640631	0.35578439
H	4.95175187	-5.10645907	-0.17623825
H	4.20787583	-2.75195956	-0.07588415
C	1.81960071	-1.84014492	0.59612667
C	1.32222009	-0.60640743	0.61863060
C	2.08654256	0.55384922	1.12467191
C	1.38665232	1.76608779	0.89801314
C	1.96398433	2.99276233	1.27842771
C	3.21972851	2.99501289	1.87245154
C	3.91028652	1.78900439	2.09888403
C	3.34894877	0.56982334	1.72668946
H	3.88014427	-0.36290770	1.89847139
H	4.89064768	1.81188351	2.56760610
H	3.67523169	3.93715036	2.16676976
H	1.43346626	3.92678017	1.11138818
H	-0.69848559	-5.22469795	2.61669853
H	-2.77579420	-3.98162521	3.15670498
H	-3.17141133	-1.74590606	2.16663441
H	-3.48127357	-0.01831219	0.25211261
H	-3.13258563	2.39961519	-0.15556480
H	-0.89046216	3.39067249	0.27056201
H	0.12466540	-0.09415361	-1.09909116

A-*trans*
42 atoms
Zero imaginary vibrational frequencies

C	0.00000000	0.00000000	0.00000000
C	0.27865856	1.42500526	-0.39995256
C	-0.76355495	2.26655471	-0.58689959
C	-2.11882171	1.73967094	-0.50467306
C	-2.40081596	0.40384163	-0.41857783
C	-1.33766806	-0.57560355	-0.39218921
C	-1.42158514	-1.92592880	-0.59520240
C	-2.59957157	-2.76433865	-0.57089550
C	-2.48796496	-4.12360480	-0.46608399
C	-1.20915351	-4.81326692	-0.36443767
C	-0.06882094	-4.11026834	-0.55136759
C	-0.16262697	-2.66682651	-0.96963386
H	-0.19236946	-2.71933466	-2.08159613
C	1.19968230	-2.07125792	-0.63960892
C	2.12195428	-3.22626704	-0.68132023
C	1.35686954	-4.42620828	-0.53431615
C	1.99302156	-5.66746982	-0.44535072
C	3.38331176	-5.73256019	-0.53800649
C	4.13420473	-4.56625617	-0.72841283
C	3.51224225	-3.31669572	-0.79778342
H	4.11153062	-2.43154741	-0.97461032
H	5.21464467	-4.63098342	-0.82769734
H	3.88462413	-6.69514536	-0.47938708
H	1.40717919	-6.57551628	-0.32530651
C	1.28033751	-0.75859893	-0.32121136
C	2.33914580	0.27064617	-0.26532998
C	1.73320280	1.55911871	-0.40264855
C	2.52263131	2.71038135	-0.47528479
C	3.90970272	2.59671889	-0.37958604
C	4.50545035	1.34229918	-0.20155597
C	3.72923367	0.18186910	-0.14567322
H	4.20976416	-0.77422235	0.02325762
H	5.58518020	1.26816396	-0.10071413
H	4.53027234	3.48781160	-0.42728804
H	2.05774780	3.68693992	-0.58554671
H	-1.20306478	-5.87298994	-0.11839931
H	-3.39260674	-4.72085902	-0.37868752
H	-3.58562705	-2.30747359	-0.53182245
H	-3.43565401	0.07458758	-0.47221519
H	-2.94120966	2.44599136	-0.59161086
H	-0.62478385	3.31982445	-0.82096611
H	-0.03509894	0.04502648	1.11221864

A-*trans* (true C_2)
42 atoms
Zero imaginary vibrational frequencies

C	0.00000000	0.00000000	0.00000000
C	2.69822092	-0.89182888	0.00000000
C	-1.42458898	-0.45079200	0.18345250
C	4.12280990	-0.44103688	0.18345250

C	-2.19213398	-0.65981206	-0.91058291
C	4.89035490	-0.23201681	-0.91058291
C	-1.58520305	-0.55006269	-2.23005706
C	4.28342397	-0.34176619	-2.23005706
C	-0.23809577	-0.41462598	-2.42468212
C	2.93631669	-0.47720290	-2.42468212
C	0.67028858	-0.36331546	-1.30088641
C	2.02793234	-0.52851342	-1.30088641
H	2.77814927	-2.00221669	-0.03107127
H	-0.07992835	1.11038781	-0.03107127
C	2.01140152	-0.58455283	1.32444380
C	0.68681941	-0.30727605	1.32444380
C	3.10812444	-0.59917630	2.31541406
C	-0.40990352	-0.29265258	2.31541406
C	4.34944365	-0.42235642	1.62598614
C	-1.65122273	-0.46947245	1.62598614
C	5.54647959	-0.30532314	2.33807649
C	-2.84825867	-0.58650574	2.33807649
C	5.52717498	-0.39651372	3.72993885
C	-2.82895406	-0.49531515	3.72993885
C	4.32124191	-0.61366502	4.40702936
C	-1.62302099	-0.27816386	4.40702936
C	3.11494952	-0.71307237	3.70899890
C	-0.41672860	-0.17875651	3.70899890
H	2.19937612	-0.91159991	4.25328349
H	0.49884481	0.01977103	4.25328349
H	4.32115390	-0.71039378	5.48974851
H	-1.62293298	-0.18143510	5.48974851
H	6.45482038	-0.31493420	4.29049114
H	-3.75659946	-0.57689468	4.29049114
H	6.48632927	-0.16400121	1.81005386
H	-3.78810835	-0.72782766	1.81005386
H	5.94120581	0.03942582	-0.83668698
H	-3.24298489	-0.93125470	-0.83668698
H	4.93225156	-0.23590028	-3.09630121
H	-2.23403064	-0.65592860	-3.09630121
H	2.53869583	-0.44333758	-3.43634170
H	0.15952509	-0.44849130	-3.43634170

6-C_2-diradical TS
42 atoms
One imaginary vibrational frequency

C	0.00000000	0.00000000	0.00000000
C	-0.37778400	-1.31880200	0.28071000
C	0.54368900	-2.18060900	0.89835700
C	1.77658500	-1.64898500	1.28960000
C	2.23221800	-0.40685900	0.86547700
C	1.30632100	0.44353900	0.23088600
H	1.61512800	1.42073400	-0.13162100
C	3.68809400	-0.15192800	0.96089900

C 4.62283900 -1.23335400 0.81101500
C 4.27140300 -2.60840000 0.61985000
C 3.91026500 -3.80938100 0.55099000
C 4.27138500 -5.20008500 0.48283500
C 3.26792700 -6.21292300 0.61300000
C 1.90929000 -5.75949300 0.69953400
C 0.77842500 -6.50732100 1.03059300
C -0.44899400 -5.87670400 1.26733100
C -0.57495900 -4.44708700 1.26800800
C 0.48564100 -3.64974600 0.93801100
C 1.70077000 -4.32738300 0.38765500
H 1.83153700 -4.08786300 -0.67709000
H -1.51179300 -4.00048500 1.59619600
H -1.30623700 -6.47684400 1.55920900
H 0.85602400 -7.58077600 1.18972100
C 3.67232900 -7.56898200 0.62967800
C 5.00679800 -7.91498000 0.51021500
C 5.99099600 -6.91678300 0.37186800
C 5.62433300 -5.57851800 0.36322100
H 6.37689500 -4.80131900 0.26557300
H 7.03686400 -7.19290400 0.26878800
H 5.29563000 -8.96279800 0.51687500
H 2.92112400 -8.34792300 0.72571200
C 6.00503000 -0.93638700 0.86029900
C 6.46715700 0.36043100 1.05187200
C 5.55473200 1.40850000 1.19333300
C 4.18783900 1.14424700 1.14289600
H 3.47973100 1.95894800 1.27000300
H 5.90508800 2.42559300 1.34765100
H 7.53625500 0.55257300 1.08636100
H 6.71268600 -1.75159300 0.74061600
H 2.43350500 -2.28190300 1.86280600
H -1.34603300 -1.69057200 -0.04517000
H -0.70791200 0.66384300 -0.49027400

6-C_s-diradical TS
42 atoms
One imaginary vibrational frequency

C 0.00000000 0.00000000 0.00000000
C -0.59726216 -1.23615883 -0.26221414
C -0.07183054 -2.06453570 -1.27325976
C 0.97884738 -1.55297245 -2.04154555
C 1.69742784 -0.41979046 -1.67950166
C 1.17335579 0.39008097 -0.65610081
H 1.70642537 1.28001019 -0.33124435
C 3.04353987 -0.26334350 -2.27251368
C 3.83477189 -1.42972050 -2.54878100
C 3.42881201 -2.77352018 -2.27050398
C 2.98119762 -3.92706455 -2.07349764
C 3.26753374 -5.32337454 -1.86141683

C 2.20743190 -6.25341277 -1.64239828
C 0.87045417 -5.71813923 -1.63660140
C -0.28875972 -6.35497349 -1.20824929
C -1.45770862 -5.60858608 -0.97094118
C -1.44035089 -4.18222402 -0.99638687
C -0.33932246 -3.49285629 -1.44757773
C 0.72181419 -4.31927642 -2.12010912
H 0.69287399 -4.25500354 -3.21844569
H -2.29267094 -3.64409389 -0.58493003
H -2.35016535 -6.11734700 -0.61724466
H -0.27761923 -7.41036507 -0.94358054
C 2.53365837 -7.61406530 -1.43803844
C 3.85162748 -8.04113397 -1.45429468
C 4.89354797 -7.12148230 -1.66751749
C 4.60027821 -5.77848264 -1.86453982
H 5.39624132 -5.05742223 -2.02636508
H 5.92627524 -7.45922966 -1.68121144
H 4.08027277 -9.09332722 -1.30467392
H 1.73838290 -8.33611699 -1.27602105
C 5.12229732 -1.25088160 -3.10567836
C 5.63428763 0.01535419 -3.36382363
C 4.86522832 1.14674947 -3.08028068
C 3.58741770 0.99827557 -2.54439868
H 2.98096284 1.87876707 -2.34777368
H 5.25484154 2.14045357 -3.28516987
H 6.63061239 0.11945088 -3.78522850
H 5.71931936 -2.13259435 -3.31957637
H 1.31868913 -2.12794318 -2.88767610
H -1.41406995 -1.58305906 0.36542455
H -0.40119580 0.62850119 0.79121197

6-C_2 DA TS
42 atoms
One imaginary vibrational frequency

C 0.00000000 0.00000000 0.00000000
C 0.35427300 1.36163800 -0.29902900
C -0.53093500 2.15671700 -1.04484000
C -1.70092900 1.60351700 -1.55114900
C -1.98957200 0.21979500 -1.37430700
C -1.18661200 -0.56913500 -0.58405300
C -1.28334600 -2.04792300 -0.35713900
C -2.18779800 -2.72716400 0.42565400
C -2.07649200 -4.13539500 0.60822700
C -0.97900800 -4.83262000 0.11815300
C 0.00781100 -4.15687600 -0.61839400
C -0.17246600 -2.76396000 -0.92529900
H 0.10209100 -2.45039600 -1.94402500
C 1.57568100 -2.12975800 -0.52803500
C 2.25097700 -3.38080500 -0.77020100
C 1.39020000 -4.53020000 -0.82745900

C 1.94758800 -5.80894400 -0.98851800
C 3.32407900 -5.95534400 -1.10486100
C 4.16894100 -4.83093600 -1.06531700
C 3.64138800 -3.55520200 -0.91136100
H 4.29775700 -2.69024700 -0.89828600
H 5.24373600 -4.96120000 -1.16111700
H 3.75247900 -6.94517200 -1.24020100
H 1.29755400 -6.67857100 -1.04572800
C 1.65233100 -0.84390300 -0.36935300
C 2.47919400 0.31110400 -0.11748000
C 1.76927600 1.55977200 -0.07019900
C 2.48200300 2.75791200 0.09997100
C 3.86453000 2.72863000 0.23288700
C 4.56109500 1.50662400 0.20183700
C 3.87860900 0.30775300 0.04084600
H 4.42037500 -0.63332900 0.03444300
H 5.64269700 1.50053300 0.30986600
H 4.41361800 3.65593400 0.37554200
H 1.94707300 3.70307100 0.15059000
H -0.84832000 -5.88410400 0.36471500
H -2.83559000 -4.65639000 1.18507400
H -3.01291000 -2.19194100 0.89029900
H -2.87036800 -0.20804700 -1.84772500
H -2.38132400 2.21732100 -2.13468800
H -0.26595900 3.18380200 -1.28657600
H 0.21203800 -0.34353800 1.02438000

6-C_2 DA TS (true C2)
42 atoms
One imaginary vibrational frequency

C 0.00000000 0.00000000 0.00000000
C -2.80494000 0.80033800 0.00000000
C 1.36610700 0.36085000 0.26588400
C -4.17104700 0.43948800 0.26588400
C 2.07263600 1.12704300 -0.67594500
C -4.87757600 -0.32670500 -0.67594500
C 1.42665100 1.59376900 -1.81381600
C -4.23159100 -0.79343100 -1.81381600
C 0.03617200 1.35568800 -2.01301700
C -2.84111200 -0.55535000 -2.01301700
C -0.66690300 0.54470000 -1.15317600
C -2.13803700 0.25563800 -1.15317600
H -2.51863200 1.83438300 0.24634800
H -0.28630800 -1.03404500 0.24634800
C -4.46732200 0.64083800 1.66715600
C 1.66238200 0.15950000 1.66715600
C -3.26634900 0.64000800 2.45649600
C 0.46140900 0.16033000 2.45649600
C -2.04971600 0.44970000 1.70544500
C -0.75522400 0.35063800 1.70544500

C	2.91149600	0.05479700	2.30001100
C	-5.71643600	0.74554100	2.30001100
C	2.98023200	-0.05886600	3.68253700
C	-5.78517200	0.85920400	3.68253700
C	1.80602800	-0.07438200	4.45796600
C	-4.61096800	0.87472000	4.45796600
C	0.55842000	0.02123500	3.85486600
C	-3.36336000	0.77910300	3.85486600
H	-0.34365900	-0.00850100	4.45872600
H	-2.46128100	0.80883900	4.45872600
H	1.87556800	-0.16743300	5.53865000
H	-4.68050800	0.96777100	5.53865000
H	3.94765000	-0.14916700	4.16987400
H	-6.75259000	0.94950500	4.16987400
H	3.81991600	0.04045500	1.70278000
H	-6.62485600	0.75988300	1.70278000
H	-5.90828400	-0.61354300	-0.47884800
H	3.10334400	1.41388100	-0.47884800
H	-4.77568300	-1.39320600	-2.53809000
H	1.97074300	2.19354400	-2.53809000
H	-2.34066700	-1.00071400	-2.86997200
H	-0.46427300	1.80105200	-2.86997200

B3LYP/6-31G(d) Thermochemistry Data (hartrees)

	E	*H*	*G*	*qG*
**6**-C_2-diradical	-999.625849	-999.244354	-999.394344	-999.391320
**6**-C_s-diradical	-999.631173	-999.249543	-999.396795	-999.394008
**A**-*trans*	-999.705330	-999.321038	-999.463903	-999.461904
**A**-*trans* (C_2)	-999.705258	-999.321039	-999.463057	-999.461003
**6**-C_2-diradical TS	-999.594091	-999.214626	-999.364245	-999.361630
**6**-C_s-diradical TS	-999.596432	-999.217150	-999.366698	-999.363982
**6**-C_2 DA TS	-999.618149	-999.238325	-999.384171	-999.381945
**6**-C_2 DA TS (C_2)	-999.618285	-999.238331	-999.382774	-999.380650
